# Comparative performance of portable DNA extraction protocols and bioinformatics workflows for rapid detection of gram-negative bacteria and antimicrobial resistance using Oxford Nanopore sequencing

**DOI:** 10.1128/spectrum.00692-26

**Published:** 2026-09-04

**Authors:** Louis Kyei-Tuffuor, Samuel Kekeli Agordzo, Angela Krobea Asante, Davina Serwaa Boateng, Winifred Nana Serwaa Adjei, Venus Nana Boakyewaa Frimpong, Rita Nartey, Francis Agyemang-Yeboah, Rea Maja Kobialka, Ahmed Abd El Wahed, Uwe Truyen, Yaw Ampem Amoako, Richard Odame Phillips, Michael Frimpong

**Affiliations:** 1 Kumasi Centre for Collaborative Research in Tropical Medicine (KCCR), Kwame Nkrumah University of Science and Technology (KNUST) 98763, Kumasi, Ghana; 2 Department of Molecular Medicine, School of Medical Sciences (SMS), Kwame Nkrumah University of Science and Technology (KNUST) 98763, Kumasi, Ghana; 3 Institute of Animal Hygiene and Veterinary Public Health, Leipzig University 9180, Leipzig, Germany; 4 Department of Medicine, School of Medical Sciences (SMS), Kwame Nkrumah University of Science and Technology (KNUST) 98763, Kumasi, Ghana; Boston Medical Center, Boston, Massachusetts, USA; Universitat Bern, Bern, Switzerland

**Keywords:** whole-genome sequencing, DNA extraction, nanopore sequencing, antimicrobial resistance, portable diagnostics, bioinformatics workflow, resource-limited settings, pathogen identification

## Abstract

Oxford Nanopore Technology (ONT) enables rapid, portable pathogen identification and antimicrobial resistance (AMR) detection, but the reliability of downstream genomic analyses is highly dependent on DNA extraction quality, particularly in resource-limited settings. This study comparatively evaluated four portable bacterial DNA extraction protocols derived from three commercial kits to determine their impact on nanopore sequencing performance, bioinformatics workflow completion, and field deployability. Six gram-negative bacterial isolates (*Escherichia coli*, *n* = 4; *Pseudomonas* sp., *n* = 1; and *Salmonella* sp., *n* = 1) were processed using four extraction protocols: SwiftX DNA, SwiftX DNA with proteinase K (ProtK), SwiftX ParaBact, and NucleoSpin Microbial. Twenty-four resulting DNA extracts were sequenced on a single multiplexed MinION R10.4.1 flow cell. Sequencing data were analyzed using validated Galaxy-based generic and species-specific pipelines. Workflow completion was defined as successful progression through quality control, assembly, virulence, plasmid, and AMR detection modules. DNA purity varied substantially by extraction protocol and was strongly associated with successful workflow completion (Kruskal–Wallis, *P* = 0.0006). Accordingly, NucleoSpin Microbial achieved 100% workflow completion, and SwiftX ParaBact achieved 83%, while both SwiftX DNA-based protocols failed to complete full workflows. Importantly, key AMR genes required to classify isolates as multidrug-resistant were consistently detected using both NucleoSpin Microbial and SwiftX ParaBact extractions. However, NucleoSpin Microbial assemblies showed significantly higher contiguity and enabled a broader, more complete detection of virulence factors, pathogenicity islands, plasmid replicons, and accessory AMR genes, reflecting enhanced genomic resolution.

**IMPORTANCE:**

Rapid whole-genome sequencing is increasingly used to detect antimicrobial resistance and guide public health responses, but its reliability depends strongly on how bacterial DNA is extracted. In this study, we have shown that DNA extraction method choice has a major impact on Oxford Nanopore sequencing performance across clinically relevant gram-negative bacteria. While silica column-based extraction maximized genomic completeness and analytical depth, paramagnetic bead-based reverse purification offered superior portability with sufficient resolution for frontline AMR surveillance. These findings highlight a practical trade-off between field deployability and high-resolution genomic characterization in low-resource settings.

## INTRODUCTION

Rapid and reliable identification of gram-negative bacterial pathogens and their antibiotic resistance (ABR) determinants remains a major challenge for clinical laboratories, particularly in low- and middle-income countries (LMICs) where molecular diagnostic capacity is limited (1, 2). Conventional culture-based methods typically require several days to yield results, delaying targeted antibacterial or antimicrobial therapy (3, 4). Although polymerase chain reaction (PCR)-based assays can shorten turnaround times, they rely on predefined targets and provide limited genomic context, thereby restricting their ability to capture the full spectrum of resistance and virulence profiles (5, 6). These limitations are especially pronounced in sub-Saharan Africa, where diagnostics for infectious diseases are largely centralized in a small number of well-resourced urban laboratories (7). This model limits access to testing in peripheral facilities and contributes to major gaps in routine AMR surveillance despite LMICs bearing a disproportionate burden of drug-resistant infections driven by highly infectious disease prevalence, widespread and often unregulated antibiotic use, and uneven access to appropriate therapies (8–11).

Recognizing this, the World Health Organization (12) identifies robust surveillance as a cornerstone of AMR control. While high-income countries benefit from established surveillance networks, many sub-Saharan African countries lack sustained national systems, resulting in fragmented or absent AMR data (13, 14). Addressing these gaps requires investment in laboratory capacity, data generation, and reporting infrastructure to support coordinated public health responses and evidence-based antimicrobial stewardship (15).

Whole-genome sequencing (WGS) offers an unbiased approach for pathogen identification, enabling comprehensive detection of AMR genes, virulence factors, sequence types, and phylogenetic relationships from a single assay (16, 17). However, widely used short-read platforms, such as Illumina HiSeq and MiSeq, despite their accuracy, are constrained by large instrument footprints, complex workflows, and extended turnaround times, which limit their suitability for decentralized or field-based use (18, 19). Portable, real-time Oxford Nanopore Technologies (ONT) devices, including the MinION, have, therefore, emerged as promising tools for rapid pathogen identification and AMR detection at or near the point of care, with several studies demonstrating culture-free bacterial diagnostics within hours (20–22). However, the performance of these nanopore-based workflows is tightly coupled to DNA extraction, particularly the recovery of long, intact fragments and the removal of inhibitory contaminants (21, 23). DNA extraction methods optimized for qPCR or short-read sequencing may yield acceptable spectrophotometric purity and concentration, yet can still perform poorly in nanopore sequencing, producing fragmented reads and low assembly contiguity that compromise downstream analyses (24, 25). This disconnect between bench-top quality metrics and real sequencing output is a major barrier to deploying rapid AMR diagnostics in the field, particularly in settings with limited resources (16).

New “reverse purification” paramagnetic-bead extraction methods, such as the SwiftX kits, are specifically designed to keep DNA fragments long and intact, even during fast, high-temperature lysis, unlike traditional silica-column protocols, which can easily break long DNA strands during the binding and elution steps (26–28). Whether these portable, paramagnetic bead-based protocols can match or outperform established silica-column kits for nanopore diagnostics in real-world field settings where portability, thermal robustness, and low per-sample costs matter most remains uncertain (27, 29, 30). To address this gap, this study comparatively evaluates four bacterial DNA extraction protocols, assessing their impact on nanopore sequencing performance, bioinformatics workflow success, and practical field-deployability in resource-limited settings.

## MATERIALS AND METHODS

### Isolate selection and sample preparation

Six bacterial isolates detailed in Table 1 were selected to evaluate the extraction protocols with Oxford Nanopore sequencing. The panel combined well-characterized reference strains and field isolates with clinically relevant resistance profiles. Two *Escherichia coli* strains from the European Reference Laboratory for Antimicrobial Resistance (EURL-AR) collection were included as external positive controls for third-generation cephalosporin and carbapenem resistance, providing stable standards for comparing extraction and sequencing performance. Specifically, this included *E. coli* TZ 3638, which has been validated by the EURL-AR to produce the carbapenemase GES-5 gene.

**TABLE 1:** Bacterial Isolates used for the extraction protocol evaluation^*a*^

Isolate ID	Description/purpose	Source
*E. coli* 2005-60-10-96-1	Validation of selective MacConkey agar plates supplemented with 1 mg/L cefotaxime, positive control (expected ESBL producer)	BfR and EURL-AR from DTU in Denmark
*E. coli* TZ 3638	Validation of selective MacConkey agar plates supplemented with 0.2 mg/L meropenem, positive control (Validated GES-5 carbapenemase producer)	BfR and EURL-AR from DTU in Denmark
PC-ATO-003-C1	VITEK-confirmed *E. coli* isolated from poultry cecal content grown on MacConkey agar plate supplemented with 1 mg/L cefotaxime (expected ESBL producer)	ADAPT-KCCR study
PC-ATO-023-C1	VITEK-confirmed *E. coli* isolated from poultry cecal content grown on MacConkey agar plate supplemented with 1 mg/L cefotaxime (expected ESBL producer)	ADAPT-KCCR study
CC-KAS-001-M1	VITEK-confirmed *Pseudomonas sp*. isolated from cattle cecal content grown on MacConkey agar plate supplemented with 0.2 mg/L meropenem (expected carbapenemase producer)	ADAPT-KCCR study
ATCC strain	ATCC *Salmonella* sp. for routine QC	KCCR Bacteriology Unit

^*a*^ EURL-AR, European Reference Laboratory for Antimicrobial Resistance; DTU, Danmarks Tekniske Universitet (Technical University of Denmark); BfR, Bundesinstitut für Risikobewertung (German Federal Institute for Risk Assessment); ATCC, American Type Culture Collection; KCCR, Kumasi Centre for Collaborative Research in Tropical Medicine; ADAPT, African One Health Network for Disease Prevention.

Two additional *E. coli* isolates and one *Pseudomonas* sp. isolate recovered from poultry and cattle caecal contents as part of the ADAPT-KCCR One Health AMR surveillance study in Ghana were included. This was to ensure that the evaluation covered diverse, field-derived, gram-negative pathogens with clinically relevant resistance profiles. These isolates were selected on MacConkey agar supplemented with cefotaxime or meropenem. An ATCC *Salmonella* sp. quality-control strain from the KCCR bacteriology unit was also included as a routine laboratory reference. This ensured that protocol performance could be evaluated across key foodborne and opportunistic gram-negative pathogens.

To establish an objective baseline for evaluating the end-to-end diagnostic workflows, the expected genomic features of the isolates were defined *a priori* based on their validated reference profiles or the selective culture media from which they were recovered. In addition to the confirmed GES-5 gene in the TZ 3638 reference strain, the expected genomic features for the field-derived *E. coli* isolates included genetic determinants for ESBL production. The *Pseudomonas* sp. isolate was expected to harbor markers for meropenem resistance. For all six isolates, stored cultures in Microbank cryovials or agar stabs were subcultured onto Columbia agar (Oxoid, United Kingdom), with 5% sheep blood and incubated at 37°C for 18–24 h. Well-isolated colonies were subcultured to confirm purity and ensure sufficient growth before downstream DNA extraction and sequencing in accordance with standard microbiological procedures (31, 32).

### Genomic DNA (gDNA) extraction methods

**Fig 1:**
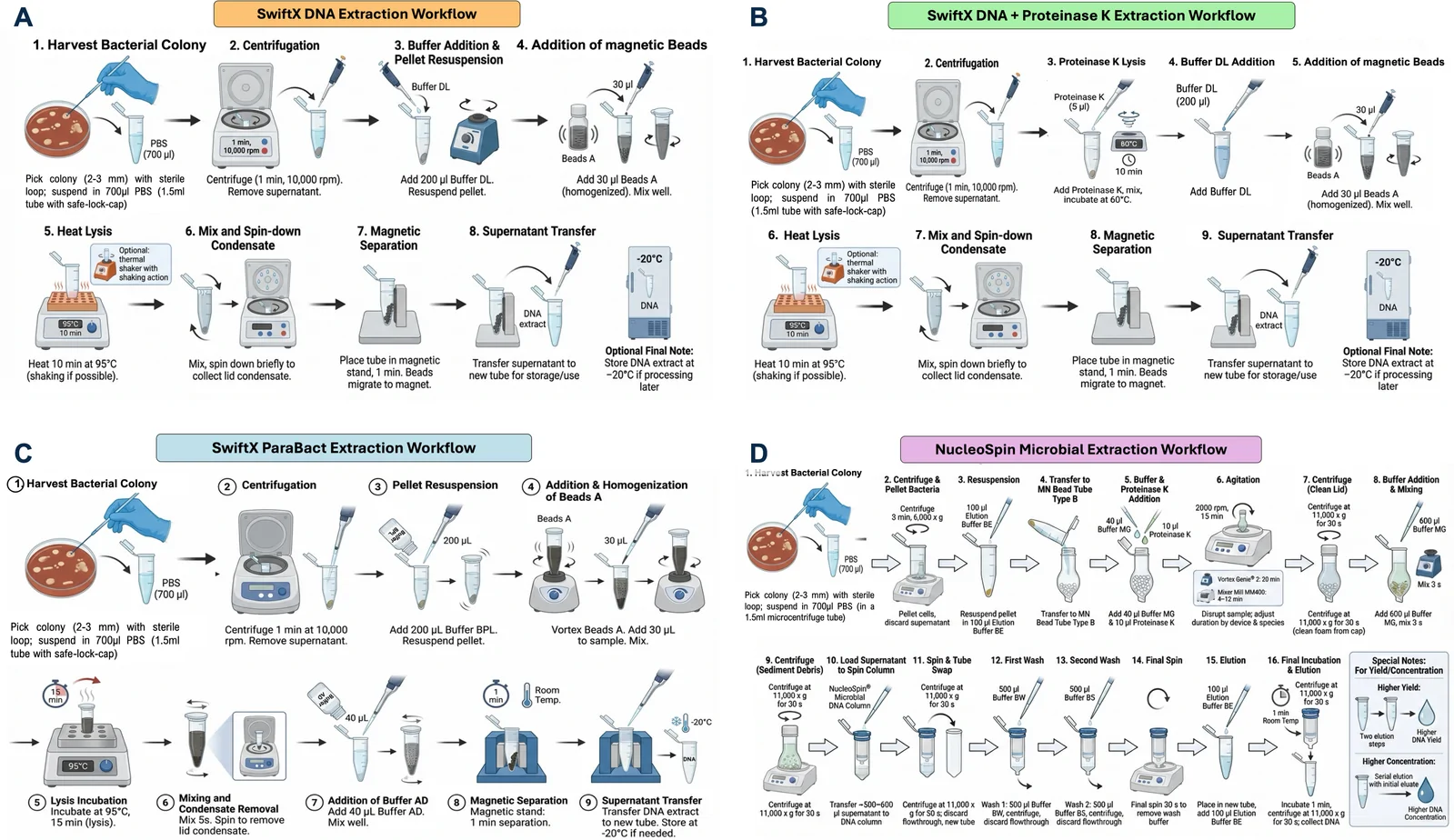
Schematic comparison of the four DNA extraction workflows evaluated. (**A**) SwiftX DNA: detergent-based lysis with reverse purification. (**B**) SwiftX DNA + ProtK: modified protocol with Proteinase K digestion. (**C**) SwiftX ParaBact: alkaline heat lysis with reverse purification. (**D**) NucleoSpin Microbial: mechanical bead-beating and enzymatic lysis, followed by silica-column purification.

To identify the most efficient protocol for bacterial DNA recovery, four extraction procedures based on three commercially available kits were evaluated across six diverse bacterial isolates. The protocols included that from the standard SwiftX DNA kit, a modified SwiftX DNA protocol incorporating Proteinase K digestion to enhance lysis of gram-negative bacteria, the SwiftX ParaBact Kit (Xpedite Diagnostics GmbH, Germany), and the NucleoSpin Microbial DNA Kit (MACHEREY-NAGEL GmbH & Co. KG, Düren, Germany), which was used as the gold-standard comparator in this study. The kits spanned distinct lysis approaches (detergent, enzymatic, alkaline heat, and mechanical bead-beating) and purification methods (paramagnetic bead-mediated reverse purification and silica membrane spin-column). A schematic summary of the evaluated extraction procedures is shown in Fig. 1.

For each isolate, genomic DNA was extracted by harvesting a standardized sweep of pure colonies (approximately 2–3 mm in diameter) with a sterile inoculation loop, which was then suspended in 700 µL of phosphate-buffered saline (Invitrogen PBS, Thermo Fisher Scientific). The SwiftX DNA protocol employed detergent-based lysis (Buffer DL) at 95°C, while the modified version incorporated a Proteinase K (ProtK) digestion step (60°C for 10 min) prior to detergent lysis to enhance recovery. The SwiftX ParaBact protocol utilized alkaline heat lysis (Buffer BPL) at 95°C. In contrast, the NucleoSpin Microbial protocol utilized mechanical disruption with glass beads and Proteinase K digestion, followed by column purification. In total, 24 gDNA extracts (six isolates × four protocols) were generated, and each method was evaluated for DNA yield, purity, and suitability for downstream Nanopore sequencing, with particular attention to robustness and field adaptability. All extractions followed the manufacturers’ instructions, with minor optimizations as detailed in File S1.

### DNA quality assessment and quantification

DNA purity was assessed by microvolume UV spectrophotometry using a DeNovix DS-11 instrument (DeNovix, Inc., Wilmington, USA). For each extract, 1.0 µL of DNA was applied to the lower pedestal; the arm was lowered to form a liquid column; and absorbance at 260 nm and 280 nm was measured in triplicate. A260/A280 ratios were recorded, and mean values were used for analysis; ratios between 1.8 and 2.0 were interpreted as indicative of acceptable DNA purity.

DNA concentration was determined fluorometrically using a Qubit 4 fluorometer with the Qubit dsDNA High-Sensitivity (HS) Assay Kit (Invitrogen, Thermo Fisher Scientific, Inc., USA). The fluorometer was calibrated with the two supplied standards, and a working solution was prepared according to the manufacturer’s instructions. For each sample, 2 µL of DNA was mixed with 198 µL of working solution in a Qubit assay tube, briefly vortexed, and incubated at room temperature for 2 min before reading. Measurements were performed in triplicate, and the instrument reported DNA concentration in ng/µL based on the standard curve.

### Whole-genome sequencing (WGS) of isolates

Whole-genome sequencing of the 24 DNA extracts was performed on the Oxford Nanopore MinION platform using the Rapid Barcoding Kit 24 V14 (SQK-RBK114.24; Oxford Nanopore Technologies, Oxford, UK) following the Nanopore-Only Microbial Isolate Sequencing Solution (NO-MISS) workflow. All 24 DNA samples were multiplexed and sequenced on a single MinION flow cell, enabling direct comparison of extraction performance under identical sequencing conditions.

For library preparation, 200 ng of purified genomic DNA per sample was used. DNA input was confirmed using a Qubit 4 fluorometer, and 200 ng in 10 µL was combined with a unique rapid barcode (RB01–RB24) and subjected to the standard 30/80°C tagmentation program according to the manufacturer’s instructions. Barcoded samples were pooled and purified using a 1:1 AMPure XP bead clean-up, washed with 80% ethanol, and eluted in elution buffer to generate a single multiplexed library.

Sequencing was performed on an R10.4.1 flow cell (FLO-MIN114) mounted on a MinION Mk1B device. Flow cell priming and library loading were carried out according to the manufacturer’s protocol. The sequencing library was prepared by combining sequencing buffer, library beads, and 12 µL of the pooled DNA library and loaded via the SpotON sample port.

The sequencing was controlled using MinKNOW software v25.05 on a laptop. The run time was set to 72 h with a minimum read length of 200 bp. High-accuracy (HAC) real-time basecalling and barcode demultiplexing (RB01–RB24) were enabled, generating POD5 and FASTQ outputs. The sequencing configuration targeted ≥50× genome coverage per sample, sufficient for downstream genome assembly, plasmid reconstruction, antimicrobial resistance profiling, and cgMLST/wgMLST analyses (24).

### Preliminary Galaxy-based generic analysis

Bioinformatic pre-processing and initial analysis were performed for all 24 Nanopore-sequenced samples on the Galaxy Europe (Galaxy.eu) platform. The gene-based pathogen identification workflow from the tutorial “Pathogen detection from (direct Nanopore) sequencing data using Galaxy-Foodborne Edition” was adapted (33, 34). This preliminary analysis was conducted before downstream species-specific bioinformatics pipelines and served to evaluate data quality, confirm taxonomy, and obtain first-pass genomic feature annotations.

Raw FASTQ reads were subjected to quality assessment using Falco v1.2.4 (35) and NanoPlot v1.46.1 (36). Adapter trimming was performed with Porechop v0.2.4 (37), and additional read filtering (minimum length and quality) was performed with fastp v1.0.1 (38). Summary reports from all pre-processing steps were collated with MultiQC v1.27 (39). Taxonomic profiling was conducted using Kraken2 v2.1.3 (40) against both the Standard + PF and Kalamari databases (41). High-quality reads were assembled *de novo* with Flye v2.9.6 (42), and assemblies were polished to consensus with Medaka v2.1.1 (43, 44).

Virulence genes were predicted with ABRicate 1.0.1 using the VFDB database (45), and antimicrobial resistance genes were detected with AMRFinderPlus v3.12.8 (46) against the National Center for Biotechnology Information (NCBI) AMR database. Putative plasmid replicons were identified by screening assemblies with PlasmidFinder v2.1.6 (47, 48) against a plasmid replicon database, and all tabular outputs were exported from Galaxy for downstream comparison of extraction protocols across isolates.

### Species-specific bioinformatics analysis

For the four *E. coli* isolates, bioinformatics analysis was performed using the Sciensano STEC Pipeline v1.2 on the Galaxy External platform, which has been validated for routine WGS-based Shiga toxin-producing *E. coli* (STEC) typing (49, 50). This “push-button” workflow carries out read trimming and basic quality control (51, 52), *de novo* assembly (42, 53, 54), and automated species confirmation (55–57), followed by integrated assays for antimicrobial resistance gene detection (46, 58), virulence gene characterization (59), O:H serotype prediction (60), plasmid replicon identification (47), and sequence typing using MLST/cgMLST schemes linked to curated public databases, such as EnteroBase and PubMLST (61–64).

The ATCC *Salmonella* quality-control isolates were analyzed with the Sciensano *Salmonella* Pipeline v1.0 on Galaxy External (62), which mirrors the structure of the STEC pipeline but implements *Salmonella*-specific assays. After read trimming, quality control, and *de novo* assembly, the pipeline performs species confirmation, *in silico* serotyping (e.g., using SISTR v1.1.1) (65, 66), antimicrobial resistance gene detection (46, 58), prediction of *Salmonella* pathogenicity islands (67, 68), virulence gene profiling (69), and plasmid replicon detection (70), again drawing on continuously synchronized reference databases to support public health surveillance applications (50, 71).

For the *Pseudomonas* sp. isolates, long-read data were re-processed on the Galaxy Europe platform using two publicly available Nanopore-optimized workflows. The Gene-Based Pathogen Identification workflow was reused for taxonomic consistency, while the Nanopore AMR Detection workflow was incorporated for dual-consensus resistance profiling. These workflows, adapted as needed, supported a final species-level characterization that included refined taxonomic identification, *de novo* assembly, and the detection of virulence and resistance determinants.

### Workflow outcome assessment

Workflow performance was evaluated by categorizing each run into three discrete outcome states based on module execution status. A workflow was considered a complete success when all analytical modules (quality control, adapter trimming, read filtering, taxonomic classification, genome assembly, antimicrobial resistance, virulence factor, and plasmid detection) were executed without premature termination. A partial success was defined as completing taxonomic assignment, with subsequent module failures in assembly, AMR, virulence, or plasmid detection. A complete failure occurred when upstream modules, such as quality control or read trimming, failed to complete.

### Sequencing and assembly metrics

Read-level metrics (total reads, mean Q-score, and read N50) were computed for all sequencing runs passing basecalling quality filters. Assembly-level metrics (assembly N50 and contig counts) were compared only for data sets generated with extraction protocols that achieved partial (assembly-only) and complete workflow success defined as uninterrupted execution through *de novo* assembly and downstream annotation modules.

### Operational feasibility

Operational feasibility was evaluated for each extraction protocol across five domains: turnaround time (TAT), equipment portability, field suitability, technical complexity, and cost. TAT was defined as the duration of the extraction procedure. Hands-on and total elapsed TAT were recorded separately for each extraction and summarized by protocol.

Equipment portability and field suitability were scored on a 1–10 scale using a standardized rubric (Table S1). For equipment portability, higher values indicated compatibility with compact, potentially battery-operated devices. Scores considered the need for high-speed versus low-speed centrifugation, dual-temperature heating requirements, the possibility of using a simple heat block in place of a thermal shaker, and any requirement for specialized accessories, such as magnetic racks. Field suitability reflected reagent storage conditions, tolerance to elevated ambient temperatures (30°C), and procedural complexity. Higher ratings were assigned to protocols that were cold chain sparing, and penalties were applied for strict dependencies, such as −20°C transport. Cost was assessed using commercial kit prices (including the Proteinase K add-on, where applicable) and calculated and reported as both the nominal cost per extraction and the effective cost per successful WGS result in US dollars.

### Statistical analysis

All statistical analyses were performed in RStudio v2025.9.2.418 (72) on a local workstation. Bioinformatics outputs were exported from Galaxy.eu and Galaxy External, organized in Microsoft Excel 365 (2025), and subsequently imported into RStudio. These statistical analyses were not integrated into the Galaxy workflow environment. Mean DNA yield and purity ratios (A260/A280) with standard deviations were calculated for each isolate and extraction protocol. Read-level metrics were compared across all extraction protocols using the Kruskal–Wallis test. In contrast, assembly contiguity metrics (assembly N50 and contig count) were compared only among protocols that produced successful assemblies for a given isolate set using the Mann–Whitney U and Dunn–Holm pairwise comparisons. The relationship between DNA purity ratios and bioinformatics workflow outcomes was assessed using a Kruskal–Wallis test, followed by Dunn’s post-hoc test with Bonferroni adjustment. Statistical significance was defined as *P* < 0.05.

## RESULTS

### DNA yield and purity across extraction protocols

Across isolates, SwiftX DNA and SwiftX DNA + ProtK produced relatively high DNA concentrations, typically ranging from 33 to 59 ng/µL. SwiftX ParaBact yielded lower concentrations, ranging from approximately 11 to 20 ng/µL. In contrast, NucleoSpin Microbial generated intermediate to high concentrations, with values up to about 100 ng/µL for *E. coli* TZ 3638 and CC-KAS-001-M1.

A260/280 purity ratios also varied by extraction protocol. SwiftX DNA and SwiftX DNA + ProtK showed ratios near 1.0–1.3 across isolates; SwiftX ParaBact produced ratios around 1.7–1.8; and NucleoSpin Microbial showed the highest and most uniform values, approximately 1.9–2.0 for all isolates. Detailed DNA yield and purity metrics for all 24 extracts are provided in Table 2.

**TABLE 2:** Comparison of mean ± SD DNA concentration and A260/280 purity ratio by extraction protocol and isolate^*a*^

Extraction protocol	Metric	Isolate					
		*E. coli* 2005-60-10-96-1	*E. coli* TZ 3638	PC-ATO-003-C1	PC-ATO-023-C1	CC-KAS-001-M1	ATCC strain
SwiftX DNA	Concentration (ng/µL)	54.3 ± 2.3	59.3 ± 0.6	58.0 ± 1.0	49.1 ± 0.8	51.0 ± 1.1	50.7 ± 0.6
	A_260/280_	1.0 ± 0.1	1.2 ± 0.0	1.2 ± 0.1	1.1 ± 0.0	1.0 ± 0.0	1.3 ± 0.0
SwiftX DNA + ProtK	Concentration (ng/µL)	41.4 ± 0.8	51.0 ± 1.0	33.5 ± 0.9	48.4 ± 1.5	52.0 ± 2.0	47.9 ± 1.1
	A_260/280_	1.1 ± 0.1	1.1 ± 0.0	1.0 ± 0.0	1.1 ± 0.0	1.0 ± 0.0	1.2 ± 0.0
SwiftX ParaBact	Concentration (ng/µL)	10.9 ± 0.2	14.9 ± 0.2	15.7 ± 0.4	11.8 ± 0.3	12.8 ± 0.3	19.7 ± 0.3
	A_260/280_	1.8 ± 0.0	1.7 ± 0.0	1.8 ± 0.0	1.8 ± 0.0	1.8 ± 0.0	1.7 ± 0.0
NucleoSpin Microbial	Concentration (ng/µL)	42.5 ± 1.0	97.5 ± 1.4	35.7 ± 0.6	48.2 ± 0.7	100.4 ± 1.4	93.6 ± 1.2
	A_260/280_	1.9 ± 0.0	1.9 ± 0.0	2.0 ± 0.0	2.0 ± 0.0	2.0 ± 0.0	1.9 ± 0.1

^*a*^ Values represent the mean ± standard deviation (SD) of DNA concentration (ng/µL) and A260/280 purity ratio from triplicate measurements for each isolate and protocol. A260/280 ratios of ~1.8–2.0 indicate high-purity DNA with minimal protein or RNA contamination.

### Sequencing performance

**Fig 2:**
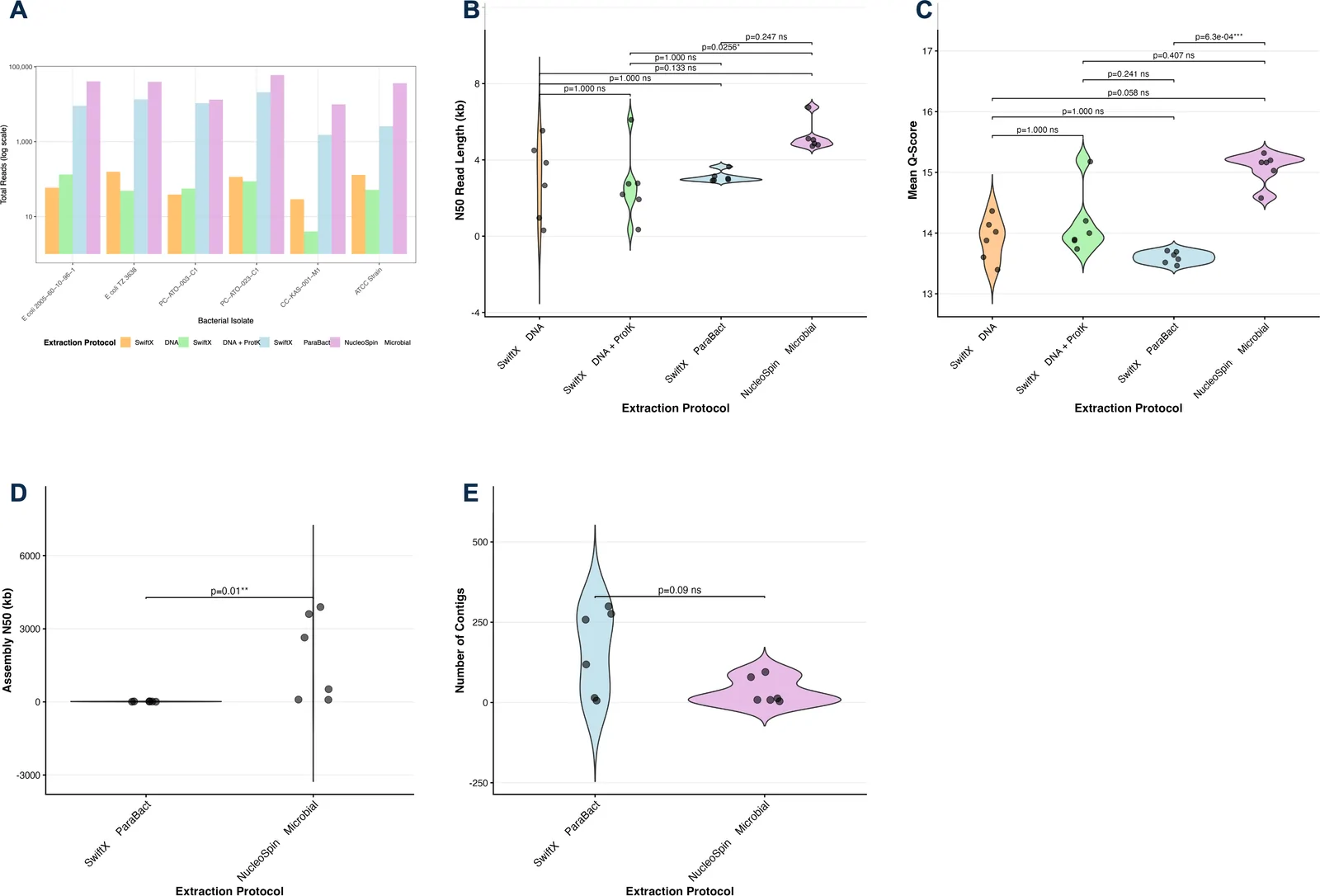
Sequencing and assembly performance metrics. (**A**) Total nanopore reads (log10 scale) per bacterial isolate for each DNA extraction protocol (SwiftX DNA, SwiftX DNA + ProtK, SwiftX ParaBact, NucleoSpin Microbial). NucleoSpin Microbial consistently yielded the highest read counts across all isolates. (**B and C**) Violin plots depicting read-level quality metrics: N50 read length (**B**) and mean Q-score (**C**) distributions across all four extraction protocols. Pairwise Dunn tests with Holm adjustment (*P*-values displayed above brackets) indicate statistically significant advantages for NucleoSpin Microbial in read quality. (**D and E**) Genome assembly contiguity metrics comparing the two protocols that successfully supported downstream assembly (SwiftX ParaBact and NucleoSpin Microbial). Panel (**D**) shows assembly N50 (kb), where NucleoSpin Microbial achieved significantly higher contiguity (*P* = 0.01), and panel (**E**) shows the number of contigs per assembly. Individual points represent assemblies from independent isolates.

The extraction protocols had a marked impact on sequencing yield and read quality (Fig. 2). For each isolate, the NucleoSpin Microbial kit yielded the highest total number of nanopore reads on a log scale (Fig. 2A). SwiftX ParaBact produced intermediate read counts, and both SwiftX DNA-based approaches yielded the lowest output, indicating clear protocol-dependent differences in sequencing efficiency.

Read length and quality distributions further favored the NucleoSpin Microbial approach. NucleoSpin Microbial showed the highest N50 read lengths (Fig. 2B) and mean Q-scores (Fig. 2C). SwiftX ParaBact produced moderately long reads with tightly clustered Q-scores. SwiftX DNA and SwiftX DNA +ProtK were characterized by shorter N50 values and lower mean Q-scores, with Dunn-Holm pairwise testing confirming significant gains in read quality and smaller but detectable gains in N50 read length for NucleoSpin Microbial compared with the SwiftX protocols.

### Species identification and taxonomic profiling

Using Kraken two for taxonomic profiling within the adapted Galaxy.eu workflow (33, 34), we achieved perfect accuracy in identifying the predefined target organisms across all 24 samples from the four extraction protocols. The target-species read proportions for *E. coli* isolates ranged from 48.2% to 72.2%, while *Pseudomonas otitidis* and *Salmonella enterica* showed ranges of 79.3% to 100% and 45.4% to 66.4%, respectively. Taxonomic classification results confirming the identity of all six isolates are listed in Table 3.

**TABLE 3:** Isolate species-level identification across protocols^*a*^

Isolate	Target species	Percentage target species			
		SwiftX DNA (%)	SwiftX DNA + ProtK (%)	SwiftX ParaBact (%)	NucleoSpin Microbial (%)
*E. coli* 2005-60-10-96-1	*Escherichia coli*	67.8	54.5	63.8	67.5
*E. coli* TZ 3638	*Escherichia coli*	68.2	59.2	64.5	70.0
PC-ATO-003-C1	*Escherichia coli*	53.9	48.2	58.3	61.9
PC-ATO-023-C1	*Escherichia coli*	72.2	55.7	60.4	64.3
CC-KAS-001-M1	*Pseudomonas otitidis*	79.3	100.0	87.0	88.6
ATCC strain	*Salmonella* *enterica*	45.4	63.5	66.4	60.3

^*a*^ Percent target species values were obtained from Kraken 2 classification reports generated by the adapted gene-based pathogen identification workflow on Galaxy.eu and represent the proportion of classified reads assigned to the expected target species for each extraction protocol–isolate combination.

### Bioinformatics workflow completion

Bioinformatics workflow completion varied substantially across extraction protocols as detailed in Table 4. Complete end-to-end analysis was achieved in 100% (6/6) of isolates processed with the NucleoSpin Microbial and 83% (5/6) with SwiftX ParaBact protocols. In contrast, both the SwiftX DNA and SwiftX DNA +ProtK protocols yielded a 0% (0/6) completion rate.

**TABLE 4:** Workflow completion rates by protocol and isolate

Extraction protocol	Isolate (*N* = 6)						Success rate, *n*/*N* (%)
	*E. coli* 2005-60-10-96-1^*a*^	*E. coli* TZ 3638^*a*^	PC-ATO-003-C1^*a*^	PC-ATO-023-C1^*a*^	CC-KAS-001-M1^*b*^	ATCC strain^*c*^	
SwiftX DNA	✗ Fail^*d*^	✗ Fail^*d*^	✗ Fail^*d*^	✗ Fail^*d*^	⚠ Partial^*e*^	✗ Fail^*d*^	0/6 (0%)
SwiftX DNA + ProtK	✗ Fail^*d*^	✗ Fail^*d*^	✗ Fail^*d*^	✗ Fail^*d*^	⚠ Partial^*e*^	✗ Fail^*d*^	0/6 (0%)
SwiftX ParaBact	✓ Complete	✓ Complete	✓ Complete	✓ Complete	⚠ Partial^*f*^	✓ Complete	5/6 (83%)
NucleoSpin Microbial	✓ Complete	✓ Complete	✓ Complete	✓ Complete	✓ Complete	✓ Complete	6/6 (100%)

^*a*^ Bioinformatics analysis performed using Sciensano STEC Pipeline v1.2 on Galaxy External.

^*b*^ Bioinformatics analysis performed using Gene-Based Pathogen Identification & Nanopore AMR workflow on Galaxy Europe (Galaxy.eu).

^*c*^ Bioinformatics analysis performed using Sciensano *Salmonella* Pipeline v1.0 on Galaxy External.

^*d*^ Failed at the onset of the analysis workflow.

^*e*^ Taxonomy-only completion: terminated prematurely after taxonomic profiling.

^*f*^ Assembly-only completion: successfully passed the* de novo* assembly stage but yielded an insufficient assembly for downstream analysis.

### Relationship between DNA purity and workflow success

**Fig 3:**
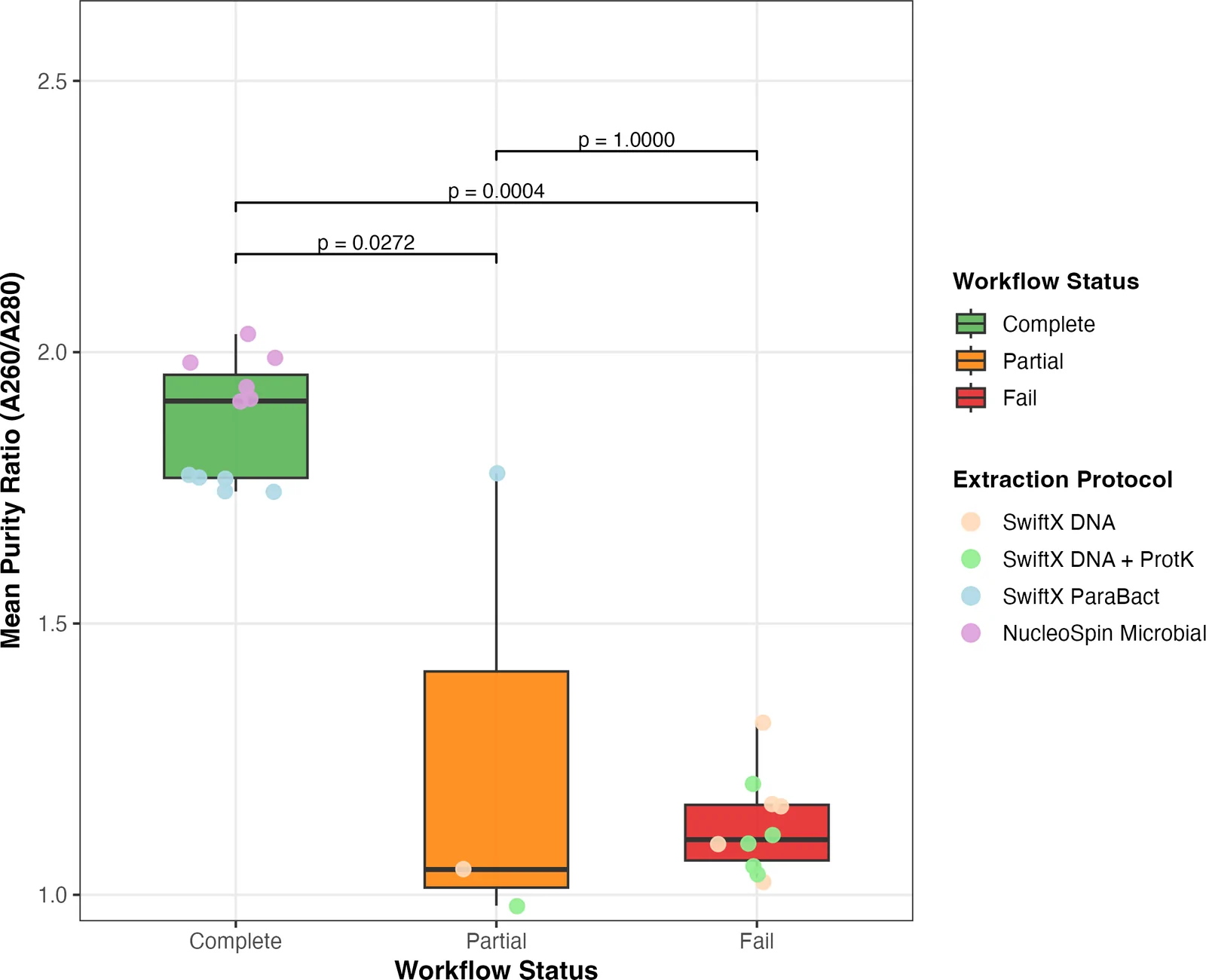
Association between DNA purity and downstream workflow success. Boxplots display the distribution of DNA purity (A260/A280 ratio) for samples categorized by their workflow status (Complete, Partial, and Fail). The solid horizontal line within each box denotes the median, while the lower and upper hinges represent the first and third quartiles, respectively, with whiskers extending to the most extreme data points. Individual sample data points are overlaid and color-coded according to the specific DNA extraction protocol utilized. Brackets above the plots indicate pairwise comparisons using Dunn’s post-hoc test with Bonferroni adjustment, with the corresponding adjusted *P*-values displayed. These results indicate that higher DNA purity was associated with successful workflow completion, with the clearest separation between complete workflows and the other two outcome categories.

Mean DNA purity (A260/A280 ratio) differed significantly across workflow outcome categories, increasing progressively from failed to completely successful workflows (Kruskal-Wallis, *P* = 0.0006; Table S2). Post-hoc Dunn’s tests with Bonferroni adjustment (Fig. 3; Table S3) showed that complete workflows had significantly higher purity ratios than both partial workflows (*P* = 0.0272) and failed workflows (*P* = 0.0004). The difference between partial and failed workflows was not statistically significant (*P* = 1.0000). As visualized in Fig. 3, failed and partially successful workflows clustered at lower median purity values (~1.0-1.3). Higher purity ratios (~1.7-2.0), however, consistently achieved complete workflows, suggesting a threshold effect.

### Failure mode characterization

Failure modes within the bioinformatics workflow were not uniformly distributed across analysis stages (Fig. S1). When initially processed through the generic Galaxy-based pipeline, data from all 24 samples, including those extracted with the SwiftX DNA and SwiftX DNA + ProtK protocols, produced sufficient reads to complete taxonomic profiling. However, when these data sets were subsequently routed to the species-specific pipelines, all six isolates from both SwiftX DNA and SwiftX DNA + ProtK protocols failed at the initial quality control and trimming stages. SwiftX ParaBact extractions, in contrast, yielded no failures during quality control, trimming, filtering, taxonomic profiling, *de novo* assembly, polishing, and detection of virulence factors, with only isolated failures observed during AMR gene and plasmid detection (Fig. S1). NucleoSpin Microbial extractions showed no failures at any stage, supporting the robustness of this protocol across the full analysis workflow.

### Performance using species-specific bioinformatics pipelines (successful protocols only)

#### Assembly contiguity

Across isolates, assemblies generated from NucleoSpin Microbial DNA showed higher N50 values than those from SwiftX ParaBact, with N50s clustering several kilobases higher than those from SwiftX ParaBact, and a significant difference between protocols (*P* = 0.01; Fig. 2D). The number of contigs per assembly overlapped across protocols, with SwiftX ParaBact exhibiting a wider spread and NucleoSpin Microbial showing a slightly lower, more compact distribution (*P* = 0.09, not significant; Fig. 2E).

#### Virulence factor characterization

Across all organism groups, the NucleoSpin Microbial protocol detected substantially higher numbers of both total and unique virulence factors than the SwiftX ParaBact protocol (Table 5). Cumulatively, NucleoSpin Microbial accounted for 318 of 373 unique virulence factors identified (85.3%) and 365 of 446 total virulence factor detections (81.8%), whereas SwiftX ParaBact recovered only 55 unique virulence factors (14.7%) and 81 total detections (18.2%). The largest differences were observed for *Pseudomonas* sp. and *Salmonella* sp., for which NucleoSpin Microbial captured nearly the entire repertoire of detected virulence factors, while SwiftX ParaBact only captured a limited subset. At the isolate level, NucleoSpin Microbial yielded higher virulence factor counts (Fig. S2) reflected by a higher mean number of detections per isolate (60.8 ± 54.5) compared to SwiftX ParaBact (13.5 ± 12.7) (Table 5). A comprehensive inventory of virulence factor genes detected by each protocol is provided in Fig. S2.

**TABLE 5:** Summary of virulence factor detection by organism group and extraction protocol^*a*^

Organism group	SwiftX ParaBact		NucleoSpin Microbial		
	Total VFs (*n*)	Unique VFs (*n*)	Total VFs (*n*)		Unique VFs (*n*)
*E. coli*	77	51	106		62
*Pseudomonas* sp.	3	3	132		132
*Salmonella* sp.	1	1	127		127
Overall detection performance					
Total unique VFs Detected (*N* = 373)	—	55 (14.7%)	—		318 (85.3%)
Total VF detections (*N* = 446)	81 (18.2%)	—	365 (81.8%)		—
Mean ± SD per isolate	13.5 ± 12.7	—	60.8 ± 54.5		—
Median per isolate (range)	11 (1-35)	—	39.5 (13–132)		—

^*a*^ VF, virulence factor; SD, standard deviation. Total VF detections represent all virulence factor genes identified per organism group and extraction protocol, including genes shared between protocols; unique VF genes represent non‑redundant virulence factor genes detected with each protocol across isolates. The percentages are calculated relative to the total number of unique virulence factors (*N* = 373) and total virulence factor detections (*N* = 446) observed across all isolates and protocols.”—”, not applicable.

#### Pathogenicity island detection

**Fig 4:**
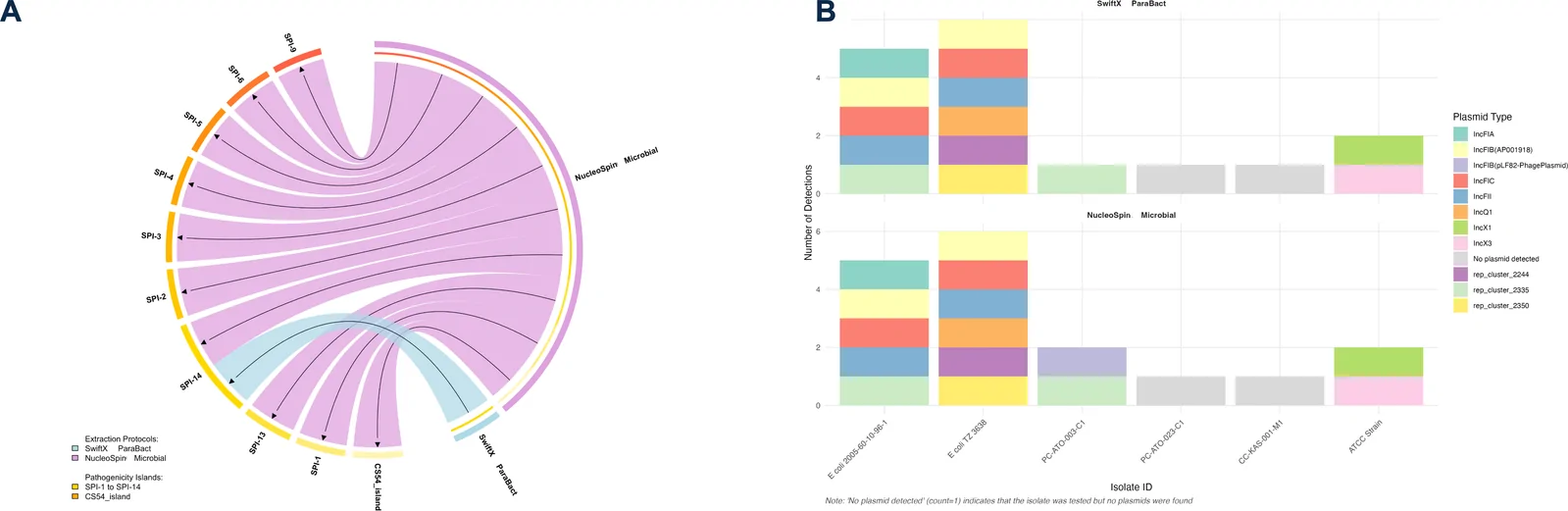
Pathogenicity island and plasmid replicon recovery using NucleoSpin Microbial and SwiftX ParaBact extraction protocols. (**A**) Chord diagram illustrating the recovery of *Salmonella* pathogenicity islands (SPI-1 through SPI-14 and the CS54 island) from isolates processed with the two successful extraction methods. Pathogenicity islands are arrayed on the left (yellow/orange arcs) and mapped against the extraction methods on the right (NucleoSpin Microbial in purple; SwiftX ParaBact in blue). NucleoSpin Microbial demonstrated broader and more consistent SPI detection, whereas SwiftX ParaBact exhibited reduced recovery across several islands. (**B**) Stacked bar charts comparing the number and diversity of plasmid replicons detected across the six bacterial isolates. The top panel displays results from the SwiftX ParaBact protocol, and the bottom panel displays results from the NucleoSpin Microbial protocol. Colors correspond to specific incompatibility (Inc) groups or replication cluster types, as defined in the legend. Gray bars indicate isolates where no plasmid markers were detected (represented as a single unit for visualization purposes).

NucleoSpin Microbial showed broader and more consistent detection of pathogenicity islands. Strong connections were observed between NucleoSpin Microbial and SPI-1 through SPI-14, indicating successful recovery of most recognized SPIs, as well as the CS54 island (Fig. 4A). The SwiftX ParaBact protocol, on the contrary, was only able to recover the SPI-14.

#### Plasmid detection

The NucleoSpin Microbial and SwiftX ParaBact protocols both demonstrated high concordance, yielding identical plasmid profiles for five of the six bacterial isolates tested (Fig. 4B). The most complex plasmid profiles were observed in the *E. coli* isolates, with both extraction methods successfully recovering high-molecular-weight incompatibility (Inc) groups. Specifically, *E. coli* 2005-60-10-96-1 exhibited five distinct plasmid markers (IncFIA, IncFIB(AP001918), IncFIC, IncFII, and rep_cluster_2235), while *E. coli* TZ 3638 displayed the highest diversity with six detected targets, including IncQ1 and multiple replication clusters (rep_cluster_2244, rep_cluster_2350). A notable variation was observed for isolate PC-ATO-003-C1. While the NucleoSpin Microbial protocol facilitated the detection of two distinct plasmid markers (rep_cluster_2244 and rep_cluster_2235), the SwiftX ParaBact method recovered only rep_cluster_2235. For the remaining isolates, results were consistent across both methods. The ATCC strain was confirmed to carry IncX1 and IncX3 plasmids, while no plasmid markers were detected in isolates PC-ATO-023-C1 or CC-KAS-001-M1 by either extraction protocol.

#### AMR gene detection

**Fig 5:**
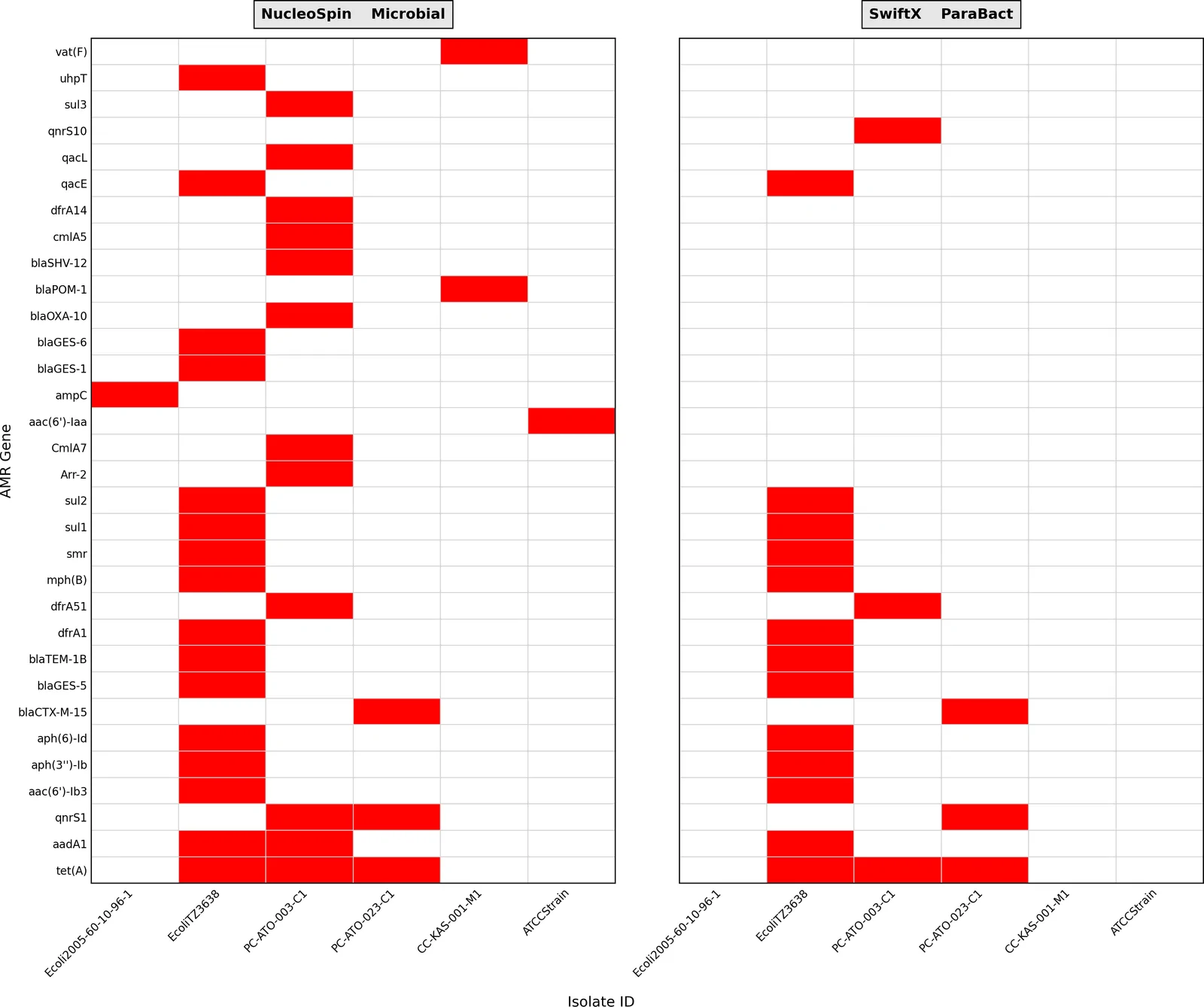
Antimicrobial resistance genes detected by each extraction protocol. Heatmap depicting presence (red) or absence (white) of AMR genes in bacterial isolates sequenced after DNA extraction with NucleoSpin Microbial (left panel) or SwiftX ParaBact (right panel); rows correspond to individual AMR genes and columns to isolates, enabling a visual comparison of resistome breadth and revealing systematically higher AMR gene detection with NucleoSpin Microbial.

Across all isolates (Fig. 5), the NucleoSpin Microbial protocol yielded a richer and more complex antimicrobial resistance (AMR) gene repertoire than SwiftX ParaBact, with numerous additional determinants detected for β-lactams (including several *blaGES* variants, *blaOXA-10*, and *blaPOM-1*), aminoglycosides (multiple *aac* and *aph* alleles), sulfonamides (*sul1–3*), trimethoprim (*dfr* genes), macrolides (*mph(B)*), and tetracycline (*tetA*). Several multidrug-resistance patterns were only observed in assemblies derived from NucleoSpin Microbial DNA protocol, whereas the corresponding SwiftX ParaBact assemblies frequently lacked these determinants or showed single-class resistance profiles. SwiftX ParaBact primarily recovered a narrower subset of AMR genes, most consistently *sul*, *dfrA*, *aadA*, and selected *aph* genes, and these were detected in fewer isolates.

### Operational performance and field-deployability assessment

The operational characteristics and field-deployability of the four DNA extraction protocols were evaluated across key criteria, including turnaround time, equipment portability, field suitability, technical complexity, and cost (Table 6). Turnaround times were shortest for SwiftX DNA (~10 min hands-on, ~15 min total), followed by SwiftX ParaBact (~10 min hands-on, ~20 min total) and SwiftX DNA + ProtK (~15 min hands-on, ~25 min total). NucleoSpin Microbial required the longest times (~25 min hands-on, ~35 min total). All protocols could be completed in less than 40 min from colony to purified DNA.

**TABLE 6:** Operational performance and field deployability assessment of DNA extraction protocols^*e*^

Criterion	SwiftX DNA	SwiftX DNA^*b*^ + ProtK	SwiftX ParaBact	NucleoSpin Microbial
1. Turnaround time				
Hands-on time (min)	~10	~15	~10	~25
Total time (min)	~15	~25	~20	~35
2. Equipment portability (1–10)				
Centrifuge requirement	Portable mini (low *g*)	Portable mini (low *g*)	Portable mini (low *g*)	Benchtop microcentrifuge (11,000 × *g*)
Thermal requirement	Heat block or shaker (shaking optional)	Two heat blocks or shakers (shaking optional)	Heat block or shaker (shaking optional)	Portable thermal shaker or heat block
Specialized equipment	Magnetic rack	Magnetic rack	Magnetic rack	None
Portability score (1–10)	9	8	9	5
3. Field suitability				
Reagent storage requirement	2–8°C preferred; up to 25°C for ≤8 weeks	−20°C (ProtK) +^*b*^ chilled transport	2–8°C preferred; 2–25°C acceptable	RT-stable kit; ProtK at 4 or −20°C after initial use
Field stability at 30°C	Excellent	Moderate	Excellent	Good
Field suitability score (1–10)	8	6	9	7
4. Technical complexity				
Training time (hours)	<2	<2	<2	<2
5. Cost analysis				
Extraction kit size (preps)	25	25	25	50
Extraction kit cost (USD)*^a^*	82.50	120.00	107.50	254.00
Per sample cost (USD)^*a*^	≈3.30	≈4.80	≈4.30	≈5.08
Cold-chain cost implication^*c*^	Low	High	Low	Moderate
Effective extraction cost per successful WGS result (USD)^*d*^	N/A (0% workflow completion rate)	N/A (0% workflow completion rate)	≈5.18 (83% workflow completion rate)	≈5.08 (100% workflow completion rate)

^*a*^ Kit costs are based on 2025 supplier quotes. Per sample cost = kit cost ÷ number of preps per kit.

^*b*^ SwiftX DNA.ProtK kit cost includes the proteinase K add-on (USD 120.00 total for 25 preps). All costs are in USD and exclude VAT, labor, equipment amortisation, and infrastructure.

^*c*^ Cold-chain cost implications reflect the additional logistical burden of refrigerated or frozen reagent storage and transport, which carries financial and operational consequences in resource-limited settings where cold-chain infrastructure is unreliable.

^*d*^ Effective extraction cost per successful WGS result = per-sample reagent cost ÷ workflow completion rate. Although SwiftX DNA-based protocols have a low nominal per-sample cost, their 0% workflow completion rate means they produce no usable WGS output, rendering the cost per result undefined. NucleoSpin Microbial and SwiftX ParaBact are directly comparable on this metric.

^*e*^ Abbreviations: ProtK: proteinase K; RT: room temperature; USD: United States dollar; VAT: value-added tax; WGS: whole-genome sequencing; N/A: not applicable.

Equipment requirements translated into high equipment-portability scores for the SwiftX kits and a lower score for NucleoSpin Microbial. SwiftX DNA, SwiftX + ProtK, and SwiftX ParaBact were all compatible with low-speed, portable mini-centrifuges and required only a simple heat block or thermal shaker and a magnetic rack. They yielded portability scores of 9, 8, and 9, respectively, on a 10-point scale. In contrast, the NucleoSpin Microbial protocol required multiple 11,000× *g* centrifugation steps on a benchtop microcentrifuge but no magnetic rack, resulting in a portability score of 5.

Reagent storage requirements were least restrictive for SwiftX ParaBact (2–25°C acceptable), resulting in a low cold-chain logistical burden. NucleoSpin Microbial features a room temperature-stable kit but requires 4 or −20°C storage for its Proteinase K component after initial use, resulting in a moderate cold-chain burden. SwiftX DNA preferred 2–8°C storage but tolerated up to 25°C for ≤8 weeks, whereas SwiftX DNA + ProtK incurred a high cold-chain logistical burden due to the strict requirement of −20°C storage and chilled transport for the Proteinase K component. At elevated ambient temperatures (30°C), field stability was rated excellent for SwiftX DNA and SwiftX ParaBact, good for NucleoSpin Microbial, and moderate for SwiftX DNA + ProtK. Overall field suitability scores (1–10 scale) were highest for SwiftX ParaBact (9), followed by SwiftX DNA (8), NucleoSpin Microbial (7), and SwiftX DNA + ProtK (6). Regarding cost, while nominal per-sample reagent prices were lowest for the SwiftX DNA (≈3.30 USD) and fairly low for the SwiftX DNA + ProtK kit (≈4.80 USD), their 0% workflow completion rate rendered their effective extraction cost per successful WGS result undefined. When adjusting for diagnostic utility, SwiftX ParaBact and NucleoSpin Microbial demonstrated highly efficient and directly comparable effective costs per successful result (≈5.18 and ≈5.08 USD, respectively). All four protocols could be implemented with minimal training, with operators requiring less than 2 hours of hands-on familiarization to perform the procedures independently.

## DISCUSSION

### DNA purity as a critical determinant of Nanopore sequencing success

This study presents a comprehensive comparison of portable DNA extraction protocols for Oxford Nanopore sequencing, explicitly linking extraction chemistry to the successful completion of downstream bioinformatics workflows. While previous studies have independently evaluated extraction yield or sequencing metrics (11, 25, 73), few have demonstrated how extraction quality directly determines the feasibility of completing clinically relevant genomic pipelines, particularly in low- and middle-income countries (LMIC) contexts.

We observed a strong and statistically significant association between DNA purity and successful completion of downstream bioinformatics workflows. Samples with purity values in the optimal range (~1.8–2.0) consistently completed all analytical stages, whereas samples with lower ratios (≤1.3) failed early, typically during quality control or shortly after taxonomic assignment. This is consistent with Oxford Nanopore Technologies’ guidance, which emphasizes high spectrophotometric purity for stable sequencing performance (74, 75).

Importantly, our findings extend this guidance by demonstrating that DNA extracts deemed acceptable by conventional spectrophotometric or qPCR-based metrics, as reported by Antane and colleagues, can still compromise nanopore read quality and hinder the completion of clinically relevant, species-specific pipelines (76). The strong association observed between DNA purity and workflow success (Kruskal–Wallis, *P* = 0.0006) indicates that purity metrics provide a practical predictor of pipeline robustness and a useful pre-sequencing screening tool. These results are particularly relevant for resource-limited settings, where early identification of suboptimal extracts could reduce failed runs, conserve sequencing resources, and improve the reliability of field-deployable genomic diagnostics (77, 78).

### Protocol-dependent differences in sequencing output and assembly quality

Sequencing performance varied significantly between extraction protocols, consistent with previous comparisons of long-read workflows and DNA isolation methods (27, 73, 79). NucleoSpin Microbial, which was the reference gold standard in this study, consistently achieved the highest read yields, longest N50 values, and highest mean Q-scores across all isolates, resulting in superior assemblies. SwiftX DNA and SwiftX DNA + ProtK designed as rapid “direct extraction” workflows with minimal purification produced very low sequencing outputs and frequent workflow failures. This is consistent with evidence that limited clean-up impairs downstream amplification and sequencing, even when lysis is efficient (80, 81).

Evaluating extraction protocols using distinct, species-specific bioinformatics pipelines introduces a potential confounding variable. It can obscure whether a workflow failure stems from the extraction chemistry or the strictness of the software. However, the failures observed with the SwiftX DNA and SwiftX DNA + ProtK protocols were fundamentally driven by inadequate DNA and sequencing data quality. While these extracts generated enough reads for basic taxonomic profiling in the generic preliminary analysis, they fell short of the stringent quality thresholds required to initiate assembly in the species-specific pipelines. Therefore, the early failure of the SwiftX DNA kits accurately reflects their inability to yield DNA of sufficient quality for high-resolution genomic analysis.

SwiftX ParaBact, despite also using paramagnetic bead-based reverse purification, performed comparably to NucleoSpin Microbial with robust read quality and assembly metrics. This suggests that the SwiftX ParaBact kit mitigates the limitations of direct extraction approaches. NucleoSpin Microbial’s combination of mechanical (glass bead beating), enzymatic lysis, and silica-column purification provided marginal additional benefits, like in other studies by Purushothaman et al. (25), for the most comprehensive recovery of virulence factors, AMR genes and plasmid regions, especially in *Pseudomonas* and *Salmonella* isolates (25).

### Practical implications for virulence factor and pathogenicity island detection

The marked differences in virulence factor and pathogenicity island recovery observed between extraction protocols (Table 5, Fig. 4A) demonstrate that DNA quality has direct consequences for how completely key pathogenicity determinants are represented in genome assemblies (82). NucleoSpin Microbial provided the most exhaustive recovery of virulence genes and *Salmonella* pathogenicity islands, supporting a detailed assessment of invasion, survival, and transmission potential, whereas SwiftX ParaBact yielded a more limited but still informative subset of these loci, sufficient for broad inference of pathogenic potential in many surveillance and diagnostic contexts.

### Plasmid detection and antimicrobial resistance gene diversity

Plasmid and AMR profiling showed that both extraction workflows recovered the clinically important plasmid backbones that commonly mobilize extended-spectrum beta-lactamases (ESBLs), carbapenemases, and other priority resistance determinants in Enterobacterales, including IncFIA, IncFIB(AP001918), IncFII, IncFIC, IncQ1, IncX1, IncX3, and several rep clusters (47, 48). This incompatibility spectrum mirrors global data, indicating that IncF and IncX plasmids dominate the dissemination of CTX-M ESBLs and NDM-type carbapenemases across clinical and food-chain settings (83, 84).

A critical discrepancy, however, emerged in the recovery of the associated antimicrobial resistance (AMR) cargo genes. The NucleoSpin Microbial revealed extensive multidrug resistance profiles, including *blaGES*, *blaPOM-1*, multiple *aac*/*aph* alleles, *sul1–3*, *dfr*, and *tetA*, whereas many of these determinants were missed with SwiftX ParaBact extraction. This “gene drop-out” phenomenon of detecting the plasmid backbone but missing the resistance gene underpins how extraction choice profoundly shapes the apparent resistome (1, 16, 85). Likely, the mechanical bead-beating and enzymatic digestion employed by the NucleoSpin Microbial kit are necessary to fully lyse cells containing high-copy resistance plasmids or preserve the integrity of specific plasmid regions during purification. Our findings align with previous studies, highlighting the critical role of combining mechanical bead beating with enzymatic lysis to achieve efficient DNA recovery (24).

Nonetheless, SwiftX ParaBact reliably captured the core IncF/IncX backbones and key resistance genes sufficient to classify isolates as multidrug-resistant, supporting its use for frontline surveillance, with referral of flagged isolates to higher-resolution workflows.

### Operational feasibility and field deployability

Despite the superior sequencing and diagnostic performance of NucleoSpin Microbial, the SwiftX protocols offer notable practical advantages for field deployment. All three SwiftX variants run on low-speed mini-centrifuges, simple heat blocks, and magnetic racks, with total extraction times of about 15–25 min versus roughly 35 min for NucleoSpin Microbial. SwiftX ParaBact reagents are stable at 2–25°C (presenting a low cold-chain burden), whereas the Proteinase K component of NucleoSpin Microbial requires refrigerated or frozen storage after initial use, introducing a moderate cold-chain constraint where infrastructure is unreliable (86). Notwithstanding these operational differences, when adjusting for workflow completion rates, both SwiftX ParaBact and NucleoSpin Microbial demonstrated highly efficient and comparable effective extraction costs per successful result (approximately 5.00 USD).

These findings highlight a practical trade-off: magnetic bead–based methods, such as SwiftX ParaBact, maximize portability and simplicity, whereas silica-column extraction with NucleoSpin Microbial maximizes diagnostic completeness and bioinformatics robustness. A realistic approach for resource-limited surveillance systems is a two-tier model using SwiftX Parabact for rapid field screening and reserving NucleoSpin for confirmatory work at reference laboratories. Future optimization of reverse-purification chemistries may narrow this performance gap.

### Strengths and limitations

The primary strengths of this study lie in its practical focus on operational feasibility in resource-limited contexts and its direct linkage of extraction performance to complete bioinformatics workflow outcomes, rather than to isolated metrics like read count alone. Furthermore, the use of a panel of clinically relevant gram-negative bacterial isolates across three distinct genera bolsters the study’s applicability. The use of NucleoSpin Microbial as a reference laboratory gold standard for analytical completeness and accuracy provides a standardized benchmark against which rapid field-deployable methods can be evaluated (87–90). Additionally, the employment of validated, species-specific bioinformatics pipelines (STEC v1.2, Salmonella v1.0, Nanopore AMR workflows) ensures the high clinical relevance of the findings (61, 91, 92).

Certain factors limited this study. The sample size was relatively small (six isolates) and restricted to three gram-negative species. Expanding this panel to include other high-priority pathogens (e.g., *Mycobacterium tuberculosis*, *Klebsiella pneumoniae*, and *Acinetobacter baumannii*) would strengthen the generalizability of these findings. Also, the evaluation was limited to three commercial extraction kits. While these were selected for their portability, accessibility, and minimal equipment requirements, this study does not represent an exhaustive market survey. For instance, conventional magnetic bead-based extraction methods were not included. Although generally simple to operate, they require extensive optimization of parameters, such as bead volume, wash cycles, elution conditions, and pipetting/mixing dynamics, to ensure consistent yield and purity (93, 94). This makes them less practical for field deployment.

A notable methodological trade-off was the multiplexing of all 24 extracts on a single MinION flow cell. While this design successfully eliminated inter-run batch effects, ensuring that observed differences were strictly attributable to the extraction protocols, it precluded the evaluation of run-to-run variability and reproducibility. Future validation studies should, therefore, include technical replicates across multiple flow cells.

Additionally, because the multi-species panel necessitated different downstream bioinformatics pipelines, perfectly isolating extraction performance from varying software stringencies proved challenging. Although preliminary generic analyses confirmed that total workflow failures were driven by poor DNA and sequencing data quality rather than software bias, pipeline variability remains a potential confounding factor. To eliminate this in future comparative studies, researchers should utilize a single-species panel evaluated through one unified, high-resolution analytical pipeline. Finally, the evaluation of reagent field stability was solely qualitative, and sequencing was performed exclusively on R10.4.1 flow cells. Future work should incorporate quantitative environmental stress testing and comparisons with newer pore generations to further contextualize these results.

### Conclusion

This comparative evaluation demonstrates that DNA extraction is a key driver of Nanopore sequencing diagnostic performance, with higher DNA purity (A260/A280 ratio) strongly associated with successful completion of the downstream bioinformatics workflow. NucleoSpin Microbial delivered 100% workflow success and the most comprehensive virulence, plasmid, and AMR profiles, making it the preferred option for centralized laboratories that require maximal diagnostic accuracy. SwiftX ParaBact provided a practical middle ground, offering high purity, an 83% workflow success rate, and the recovery of core virulence and resistance determinants suitable for many surveillance applications. Although SwiftX DNA-based protocols failed at assembly and detailed AMR profiling, they consistently supported correct taxonomic profiling and, therefore, retain value for rapid species-level screening. Programs planning nanopore-based AMR surveillance, particularly in resource-limited settings, must prioritize the careful selection and optimization of extraction workflows. Future work should focus on improved or hybrid bead/column chemistries that successfully combine field portability with high analytical performance.

## Supplementary Material

Reviewer comments

## Data Availability

The raw sequencing data generated and analyzed during the current study are available in the NCBI Sequence Read Archive under BioProject accession number PRJNA1492751.

## SUPPLEMENTAL MATERIAL

Supplemental material file S1 spectrum.00692-26-s0001.pdf Detailed extraction protocols.

Supplemental material file S2 spectrum.00692-26-s0002.xlsx Comprehensive inventory of virulence factor genes detected by each protocol.

Supplemental material file S3 spectrum.00692-26-s0003.docx Figs. S1 and S2.

Supplemental material file S4 spectrum.00692-26-s0004.pdf Tables S1 to S3.

OPEN PEER REVIEW reviewer-comments.pdf An accounting of the reviewer comments and feedback.

ASM does not own the copyrights to Supplemental Material that may be linked to, or accessed through, an article. The authors have granted ASM a non-exclusive, world-wide license to publish the Supplemental Material files. Please contact the corresponding author directly for reuse.
