# Supplementary material for: Comparative performance of portable DNA extraction protocols and bioinformatics workflows for rapid detection of gram-negative bacteria and antimicrobial resistance using Oxford Nanopore sequencing
Source: Microbiol Spectr. 2026 Sep 4;14(10):e00692-26. doi: 10.1128/spectrum.00692-26 (PMC13689490; doi:10.1128/spectrum.00692-26)
Supplement: Supplementary file 1 [file reviewer-comments.pdf]

ASM | Microbiology Spectrum

Comparative Performance of Portable DNA Extraction Protocols and Bioinformatics Workflows for Rapid Detection of Gram-Negative Bacteria and Antimicrobial Resistance Using Oxford Nanopore Sequencing

Louis Kyei-Tuffuor, Samuel Agordzo, Angela Asante, Davina Boateng, Winifred Adjei, Venus Frimpong, Rita Nartey, Francis Agyemang-Yeboah, Rea Maja Kobialka, Ahmed Abd El Wahed, Uwe Truyen, Yaw Amoako, Richard Phillips, and Michael Frimpong

Corresponding Author(s): Michael Frimpong, Kwame Nkrumah University of Science and Technology

Review Timeline:

Submission Date:	March 2, 2026
Editorial Decision:	June 9, 2026
Revision Received:	July 14, 2026
Accepted:	August 5, 2026

Editor: Marisa Nielsen

Reviewer(s): Disclosure of reviewer identity is with reference to reviewer comments included in decision letter(s). The following individuals involved in review of your submission have agreed to reveal their identity: Alban Ramette (Reviewer #3)

Transaction Report:

(Note: With the exception of the correction of typographical or spelling errors that could be a source of ambiguity, letters and reports are not edited. The original formatting of letters and referee reports may not be reflected in this compilation.)

DOI: https://doi.org/10.1128/spectrum.00692-26

Re: Spectrum00692-26 (Comparative Performance of Portable DNA Extraction Protocols and Bioinformatics Workflows for Rapid Detection of Pathogens and Antimicrobial Resistance Using Oxford Nanopore Sequencing.)

Dear Dr. Michael Frimpong:

Thank you for the privilege of reviewing your work. Below you will find my comments, instructions from the Spectrum editorial office, and the reviewer comments.

Please return the manuscript within 60 days; if you cannot complete the modification within this time period, please contact me. If you do not wish to modify the manuscript and prefer to submit it to another journal, notify me immediately so that the manuscript may be formally withdrawn from consideration by Spectrum.

Revision Guidelines
To submit your modified manuscript, log into the submission site at https://spectrum.msubmit.net/cgi-bin/main.plex. Go to Author Tasks and click the appropriate manuscript title to begin. The information you entered when you first submitted the paper will be displayed; update this as necessary. Note the following requirements:

• Upload point-by-point responses to the issues raised by the reviewers in a file named "Response to Reviewers," NOT in your cover letter.
• Upload a compare copy of the manuscript (without figures) as a "Marked-Up Manuscript" file.
• Upload a clean .DOC/.DOCX version of the revised manuscript and remove the previous version.
• Each figure must be uploaded as a separate, editable, high-resolution file (TIFF or EPS preferred), and any multipanel figures must be assembled into one file.
• Any supplemental material intended for posting by ASM should be uploaded with their legends separate from the main manuscript. You can combine all supplemental material into one file (preferred) or split it into a maximum of 10 files with all associated legends included.

For complete guidelines on revision requirements, see our Submission and Review Process webpage. Submission of a paper that does not conform to guidelines may delay acceptance of your manuscript.

Data availability: ASM policy requires that data be available to the public upon online posting of the article, so please verify all links to sequence records, if present, and make sure that each number retrieves the full record of the data. If a new accession number is not linked or a link is broken, provide Spectrum production staff with the correct URL for the record. If the accession numbers for new data are not publicly accessible before the expected online posting of the article, publication may be delayed; please contact production staff (Spectrum@asmusa.org) immediately with the expected release date.

Publication Fees: For information on publication fees and which article types are subject to charges, visit our website. If your manuscript is accepted for publication and any fees apply, you will be contacted separately about payment during the production process; please follow the instructions in that e-mail. Arrangements for payment must be made before your article is published.

ASM Membership: Corresponding authors may join or renew ASM membership to obtain discounts on publication fees. Need to upgrade your membership level? Please contact Customer Service at Service@asmusa.org.

The ASM Journals program strives for constant improvement in our submission and publication process. Please tell us how we can improve your experience by taking this quick Author Survey.

Thank you for submitting your paper to Spectrum.

Sincerely,
Marisa Nielsen
Editor
Microbiology Spectrum

Reviewer #2 (Comments for the Author):

The authors describe the results of studies to assess the performance of four DNA extraction methods for use with Nanopore sequencing. The study question is important and has practical implication. The experimental approach is scientifically sound. The results are novel and informative. In general, the authors' conclusions are adequately supported by the presented information.

Major comments:
The manuscript could be significantly improved by incorporating the many of the supplemental figures and tables in the main text. It is important for the authors to show the results in the Results Section and not simply summarize the findings.
The cost analysis is superficial and misses many factors that are known to influence the cost of a test. The authors should conduct and report a thorough cost analysis.
The description of the operational characteristics is useful, However, the numerical scores assigned to the various factors is subjective and no criteria were described for assigning a specific score to a factor. It is not obvious how one would interpret the numerical scores. The authors should explain the criteria that wee used to assign the scores.
Minor comment:
Line 682. It is not clear that three gram-negative bacterial species is a "phylogenetically diverse bacterial panel".

Reviewer #3 (Public repository details (Required)):

The sequencing data has not been deposited in a public repository. At least this information is not indicated in the manuscript or accompanying files.

Reviewer #3 (Comments for the Author):

This research paper evaluates four portable DNA extraction protocols to determine their effectiveness for rapid pathogen identification and antimicrobial resistance (AMR) surveillance using Oxford Nanopore sequencing. Findings indicate that DNA purity is a critical predictor of success, as higher quality extracts led to more reliable bioinformatics workflow completion and greater genomic resolution. Ultimately, the authors highlight a necessary trade-off between analytical depth and field deployability in resource-limited settings. This work provides a framework for selecting extraction methods that support decentralized genomic diagnostics in public health monitoring.
MAJOR COMMENTS
1) The manuscript's stated focus is on the rapid detection of "pathogens," but the experimental design exclusively utilizes Gram-negative bacteria. Specifically, the study tests the protocols using 4 Escherichia coli, 1 Pseudomonas sp., and 1 Salmonella sp. isolates. The broad use of the word "pathogens" may thus be misleading, as the results may not translate to Gram-positive bacteria, fungi, or other pathogen classes. The title should be revised to specify "Gram-negative bacteria" to be more precise and reflect the true scope of the study.
2) The study design suffers from an unequal distribution of replicates across the selected species, utilizing four E. coli isolates, but only one Pseudomonas sp. isolate and one Salmonella sp. isolate. This significant imbalance skews the dataset heavily toward E. coli and limits the statistical robustness and generalizability of the findings for the other species evaluated. In addition, the study evaluates the extraction protocols with a lack of experimental replication, as all 24 DNA extracts were multiplexed and sequenced on a single MinION R10.4.1 flow cell. Because only one flow cell was used for the entire experimental run, the study cannot adequately assess run-to-run variability, reproducibility, or the overall robustness of the sequencing workflows, which are all key elements for reliability and field deployability.
3) While the study incorporates well-characterized reference strains, such as E. coli isolates from the European Reference Laboratory for Antimicrobial Resistance and an ATCC Salmonella sp. quality-control strain, the authors fail to describe the specific expected genomic features of these strains. Without defining the baseline genomic metrics, such as precise genome size, specific sequence types, or expected resistance gene and plasmid profiles, it is difficult to make an objective, quantitative comparison of the assemblies against a true reference genome.
4) There is a significant contradiction in the described methodology regarding the DNA extraction procedure. In one section, the authors state that isolates were subcultured to "generate sufficient biomass for downstream DNA extraction", but they subsequently specify that genomic DNA was extracted from "a single bacterial colony suspended in 700 µL of PBS". This discrepancy between requiring generalized "sufficient biomass" versus specifically picking one colony makes the exact extraction procedure unclear and could hinder the reproducibility of the reported yields. Line 354 and onwards report rather large DNA concentrations if the genomic isolation was done on one bacterial colony each time.
5) The use of different, species-specific bioinformatics pipelines introduces a confounding variable when assessing workflow performance. E. coli isolates were processed with the Sciensano STEC Pipeline, Salmonella with the Sciensano Salmonella Pipeline, and Pseudomonas with generic workflows. Consequently, when a pipeline fails entirely-as occurred with the SwiftX DNA-based protocols-it is not entirely clear whether the failure is due to general, systematic data quality issues or because of the strict constraints and species-specific thresholds of the individualized software treatments.
6) The study evaluates the relationship between DNA purity and workflow success in Figure 3, but may have used an inappropriate statistical test. The purity ratio is a continuous quantitative variable, while workflow success is defined by three discrete categories: Fail, Partial, and Complete. Assessing this relationship with a linear trendline and Spearman's rank correlation coefficient may not be the most statistically robust or appropriate method for comparing a quantitative variable against categorical data.
MINOR COMMENTS
1) The formatting and layout of Figures 1 and 2 are too small to be easily read and interpreted. Figure 1 contains four separate schematic workflows, and Figure 2 includes five different graphical panels evaluating sequencing metrics. The authors should consider reorganizing these multi-panel visual elements-such as placing the four panels of Figure 1 into a single column-to

ensure that the details, text, and axes are legible.
2) Line 247 when defining the sequencing configuration, the authors state that they targeted "{greater than or equal to}50× genome coverage per sample," which they claim is sufficient for downstream assembly and antimicrobial resistance profiling. Please provide a formal reference to justify or support the use of this specific coverage threshold for Oxford Nanopore data.
3) In the methodology section detailing the statistical analyses (line 340), the authors note that all statistics were computed in R Studio. Because the study relies heavily on the Galaxy.eu platform for its bioinformatics workflows, the manuscript does not make it clear whether these R analyses were also integrated into the Galaxy environment or performed separately.

Please find our point-by-point response below. Changes are highlighted in yellow in the revised manuscript and tracked.

Reviewer comments are reproduced in italics, followed by our responses and a description of the corresponding changes made in the revised manuscript.

Reviewer #2

General Comment
"The authors describe the results of studies to assess the performance of four DNA extraction methods for use with Nanopore sequencing. The study question is important and has practical implication. The experimental approach is scientifically sound. The results are novel and informative. In general, the authors' conclusions are adequately supported by the presented information."

Response: We thank Reviewer #2 for the positive assessment of the study design, novelty, and overall conclusions. We have carefully considered all major and minor comments and have revised the manuscript accordingly, as detailed below.

Major Comment 1
"*The manuscript could be significantly improved by incorporating the many of the supplemental figures and tables in the main text. It is important for the authors to show the results in the Results Section and not simply summarize the findings."*

Response: We agree with the reviewer that key data should be visible in the main text. In the revised manuscript, we have moved the most informative supplementary tables and figures into the main Results section and simplified the remaining supplementary materials to avoid redundancy. Specifically:

- The table summarizing DNA yield and purity for all 24 extracts (previously Supplementary Table S1) has been moved into the results section as Table 2 and is now referenced in Section 3.1 (DNA yield and purity across extraction protocols).

- The table summarizing taxonomic classification results for all isolates (previously Supplementary Table S2) has been moved into the results section as Table 3 and is referenced in Section 3.3 (Species identification and taxonomic profiling).

- The table summarizing workflow completion outcomes by protocol and isolate (previously Supplementary Table S3) has been moved into the results section as Table 4 and is referenced in Section 3.4 (Bioinformatics workflow completion).

- The table summarizing virulence factor detection by organism group and protocol (previously Supplementary Table S4) has been moved into the results section as Table 5 and is referenced in Section 3.7.2 (Virulence factor characterization).

- Supplementary figures illustrating pathogenicity island detection (Figure S5) and plasmid detection (Figure S6) have been integrated into the main results as a new multipaneled Figure 4.

Major Comment 2
"The cost analysis is superficial and misses many factors that are known to influence the cost of a test. The authors should conduct and report a thorough cost analysis."

Response: We thank the reviewer for this important comment. We completely agree that factors such as personnel, sequencing consumables, and bioinformatics infrastructure significantly influence the overall cost of a diagnostic test. However, because our shared, multiplexed, single-flow-cell design meant these downstream costs were identical across all experimental groups, our economic analysis was explicitly designed to isolate the differential costs associated solely with the varying extraction protocols. Including the downstream constants would inflate the per-sample estimates without providing additional discriminatory value for comparing the kits.
Nevertheless, we acknowledge that our initial analysis was too narrowly focused. To directly address your concern and improve transparency, we have substantially expanded our cost evaluation in the revised Table 6 (formerly Table 2) to include:

i. Cold-chain cost implications: A qualitative assessment of the logistical and financial consequences of transporting refrigerated or frozen reagents in resource-limited settings.
ii. Effective extraction cost per successful WGS result: A new quantitative metric calculated by dividing the nominal per-sample extraction cost by the observed workflow completion rate. This provides a much more accurate reflection of true diagnostic utility while strictly maintaining our methodological boundary of excluding shared downstream sequencing costs.

We have also clarified in the Methods (section 2.9) and Table 6 footnote that the reported costs represent extraction-specific costs and not a comprehensive economic assessment of the entire sequencing workflow.

Major Comment 3
"*The description of the operational characteristics is useful. However, the numerical scores assigned to the various factors is subjective and no criteria were described for assigning a specific score to a factor. It is not obvious how one would interpret the numerical scores. The authors should explain the criteria that wee used to assign the scores."*

Response: We agree with the reviewer. We have added a comprehensive scoring rubric in the supplementary materials (now Table S1) and summarised it in the Methods section. This rubric explicitly details the point allocations to objectively justify how the portability and field-suitability scores out of 10 were calculated.

Minor Comment
"Line 682. It is not clear that three gram-negative bacterial species is a 'phylogenetically diverse bacterial panel'".

Response: We thank the reviewer for highlighting this imprecise wording. We have revised the statement in the Discussion (Section 4.6, Strengths and limitations) to: "a panel of clinically relevant Gram-negative bacterial isolates representing three distinct genera in line 982".

Reviewer #3

Public Repository Comment
"*The sequencing data has not been deposited in a public repository. At least this information is not indicated in the manuscript or accompanying files."*

Response: We have now uploaded all raw sequencing data (FASTQ files) to the NCBI

Sequence Read Archive (SRA) under BioProject accession number. This information has been added to the Data Availability Statement.

Major Comment 1

The manuscript's stated focus is on the rapid detection of "pathogens," but the experimental design exclusively utilizes Gram-negative bacteria. Specifically, the study tests the protocols using 4 E. coli, 1 Pseudomonas sp., and 1 Salmonella sp. isolates. The broad use of the word "pathogens" may thus be misleading, as the results may not translate to Gram-positive bacteria, fungi, or other pathogen classes. The title should be revised to specify "Gram-negative bacteria" to be more precise and reflect the true scope of the study."

Response: We agree with the reviewer's observation. We have revised the title to: "Comparative Performance of Portable DNA Extraction Protocols and Bioinformatics Workflows for Rapid Detection of Gram-Negative Bacteria and Antimicrobial Resistance Using Oxford Nanopore Sequencing." The manuscript text has also been reviewed throughout to replace broad references to "pathogens" with more precise terminology where appropriate.

Major Comment 2

"The study design suffers from an unequal distribution of replicates across the selected species, utilizing four E. coli isolates, but only one Pseudomonas sp. isolate and one Salmonella sp. isolate. This significant imbalance skews the dataset heavily toward E. coli and limits the statistical robustness and generalizability of the findings for the other species evaluated. In addition, the study evaluates the extraction protocols with a lack of experimental replication, as all 24 DNA extracts were multiplexed and sequenced on a single MinION R10.4.1 flow cell. Because only one flow cell was used for the entire experimental run, the study cannot adequately assess run-to-run variability, reproducibility, or the overall robustness of the sequencing workflows, which are all key elements for reliability and field deployability."

Response: We thank the reviewer for identifying these limitations, which are valid and important.

Regarding the unequal species distribution: The imbalance reflects the practical constraints of our study context. The four *E. coli* isolates included two well-characterized EURL-AR reference strains serving as positive controls for ESBL and carbapenem resistance, and two field-derived isolates from our One Health surveillance study, providing both reliable reference points and real-world variety. The single *Pseudomonas* sp. and *Salmonella* sp. isolates were the only available VITEK-confirmed strains with clinically relevant resistance profiles at the time of the study. We acknowledge this imbalance explicitly as a limitation in Section 4.6 and note that it restricts species-level generalizability for *Pseudomonas* and *Salmonella*.

Regarding single flow cell use: While cost constraints in our resource-limited setting precluded multiple independent sequencing runs, this single-run design provided an inherent methodological advantage for this comparative study. By multiplexing all 24 samples head-to-head on a single flow cell, we effectively controlled for inter-run batch effects, ensuring that any observed differences in sequencing yield, quality, and workflow outcomes are strictly attributable to the DNA extraction protocol rather than flow-cell-to-flow-cell variation. We have clarified this rationale in Section 2.4 and formally acknowledged the limitation in Section 4.6 by adding the following text: "The study utilized a single MinION flow cell. While this approach effectively eliminates batch effects, it prevents the assessment of run-to-run variability. To confirm reproducibility, future studies must incorporate technical replicates across multiple flow cells." – line 1066

Major Comment 3

"While the study incorporates well-characterized reference strains, such as E. coli isolates from the European Reference Laboratory for Antimicrobial Resistance and an ATCC Salmonella sp. quality-control strain, the authors fail to describe the specific expected genomic

features of these strains. Without defining the baseline genomic metrics, such as precise genome size, specific sequence types, or expected resistance gene and plasmid profiles, it is difficult to make an objective, quantitative comparison of the assemblies against a true reference genome."

Response: We appreciate the reviewer raising this important point regarding baseline metrics. A primary goal of this study was to evaluate these end-to-end diagnostic workflows using unsequenced isolates to accurately simulate a real-world diagnostic deployment. Because of this, prior whole-genome sequence data did not exist for most of the panel before our sequencing efforts. However, we agree that defining expected features for our reference strains strengthens the evaluation. We have updated the manuscript to clearly state that the *E. coli* TZ 3638 reference strain harbours the carbapenemase GES-5 gene, as independently validated by the EURL-AR – (line 151). For the field isolates, we established an objective phenotypic baseline using selective screening to evaluate the accuracy of the downstream bioinformatic pipelines. Based on the selective culture plates from which the isolates were recovered (detailed in Table 1), the expected genomic features for the field *E. coli* isolates included genetic determinants for ESBL production, while the *Pseudomonas* sp. isolate was expected to harbour markers for meropenem resistance. We also confirmed the species identity of these field isolates using the VITEK 2 COMPACT automated biochemical testing system. We have made the necessary adjustments in the revised manuscript to state clearly these expected genomic features and baseline metrics.

Major Comment 4
"There is a significant contradiction in the described methodology regarding the DNA extraction procedure. In one section, the authors state that isolates were subcultured to "generate sufficient biomass for downstream DNA extraction", but they subsequently specify that genomic DNA was extracted from "a single bacterial colony suspended in 700 µL of PBS". This discrepancy between requiring generalized "sufficient biomass" versus specifically picking one colony makes the exact extraction procedure unclear and could hinder the reproducibility of the reported yields. Line 354 and onwards report rather large DNA concentrations if the genomic isolation was done on one bacterial colony each time."
Response: We thank the reviewer for highlighting the need for clearer methodological descriptions. The phrase "sufficient biomass" was intended to describe achieving robust, sufficient growth on the subculture plate before extraction, rather than defining the extraction input itself. To ensure consistent input across protocols, we standardized sampling by harvesting a sweep of pure colonies (approximately 2–3 mm in diameter) with a sterile inoculation loop, rather than picking a single colony. We have revised the Methods section (line 211) (and Supplementary File S1) to state these procedures appropriately.

Major Comment 5
"The use of different, species-specific bioinformatics pipelines introduces a confounding variable when assessing workflow performance. E. coli isolates were processed with the Sciensano STEC Pipeline, Salmonella with the Sciensano Salmonella Pipeline, and Pseudomonas with generic workflows. Consequently, when a pipeline fails entirely-as occurred with the SwiftX DNA-based protocols-it is not entirely clear whether the failure is due to general, systematic data quality issues or because of the strict constraints and species-specific thresholds of the individualized software treatments."

Response: We thank the reviewer for highlighting this important consideration. The use of species-specific pipelines was intentional. Our objective was to evaluate protocol performance under realistic end-user conditions where validated, species-specific workflows are routinely employed. The reviewer raises a valid concern that different pipeline thresholds introduce a potential confounding variable. However, we have clear evidence demonstrating that the failure of the SwiftX DNA-based protocols was driven by inadequate DNA and sequencing data quality rather than an inherent bias in the software. As detailed in the manuscript, all 24

samples were initially processed through a generic Galaxy-based pipeline. In this unified workflow, the samples processed using the SwiftX DNA and SwiftX DNA + ProtK protocols produced sufficient reads to reach the taxonomic profiling stage. However, the overall sequencing data was of fundamentally poor quality. When these datasets were subsequently fed into the highly stringent species-specific pipelines, they failed at the very onset. This immediate failure occurred because the sequencing data generated using the SwiftX DNA protocols could not meet the strict minimum baseline thresholds for depth of coverage, read length, and quality scores demanded by the software even to initiate *de novo* assembly. Notably, samples from the SwiftX ParaBact and NucleoSpin Microbial protocols utilized the same species-specific pipelines and completed the workflows. This confirms that the software constraints function correctly with adequate input data. The early failure of the SwiftX DNA-based protocols accurately reflects the biological reality. These extraction protocols failed to yield DNA of sufficient quality for high-resolution genomic analysis, and this deficiency was properly flagged by the stringent quality gates of the bioinformatics software. To address this concern and ensure complete transparency, we have expanded the Results (Section 3.6)-(line 610) and Discussion (Sections 4.2 (line 887) and 4.6 (1077)). These revisions fully acknowledge the confounding variable and clearly articulate why poor data quality was the primary driver of these workflow failures.

Major Comment 6
"The study evaluates the relationship between DNA purity and workflow success in Figure 3 but may have used an inappropriate statistical test. The purity ratio is a continuous quantitative variable, while workflow success is defined by three discrete categories: Fail, Partial, and Complete. Assessing this relationship with a linear trendline and Spearman's rank correlation coefficient may not be the most statistically robust or appropriate method for comparing a quantitative variable against categorical data."

Response: We thank the reviewer for the statistical insight. We have removed the linear trendline and Spearman's rank correlation. Instead, we now employ a Kruskal-Wallis test, followed by a post-hoc Dunn's test to properly compare the continuous distribution of purity ratios across the three discrete categorical workflow outcomes. Figure 3 has been updated to a box plot to display these group differences accurately, and the Statistical Methods and Results sections have been updated accordingly.

Minor Comment 1
"The formatting and layout of Figures 1 and 2 are too small to be easily read and interpreted. Figure 1 contains four separate schematic workflows, and Figure 2 includes five different graphical panels evaluating sequencing metrics. The authors should consider reorganizing these multi-panel visual elements-such as placing the four panels of Figure 1 into a single column-to ensure that the details, text, and axes are legible."

Response: Figures 1 and 2 have been reformatted to improve readability. Panel sizes, font sizes, axis labels, and figure layout have been adjusted to enhance clarity.

Minor Comment 2
"Line 247 when defining the sequencing configuration, the authors state that they targeted '{greater than or equal to}50× genome coverage per sample,' which they claim is sufficient for downstream assembly and antimicrobial resistance profiling. Please provide a formal reference to justify or support the use of this specific coverage threshold for Oxford Nanopore data."

Response: We thank the reviewer for this request. The 50× coverage threshold was selected based on Oxford Nanopore Technologies' own input QC guidance and empirical evidence from the published literature demonstrating that ≥50× coverage is sufficient for accurate *de novo* assembly and resistance gene detection with long-read nanopore data. We have added

supporting references to Section 2.4.

Minor Comment 3
"In the methodology section detailing the statistical analyses (line 340), the authors note that all statistics were computed in R Studio. Because the study relies heavily on the Galaxy.eu platform for its bioinformatics workflows, the manuscript does not make it clear whether these R analyses were also integrated into the Galaxy environment or performed separately."

Response: We thank the reviewer for this request for clarification. All statistical analyses described in Section 2.10 were performed independently in R Studio v2025.9.2.418 on a local workstation and were not integrated into the Galaxy.eu environment. Galaxy was used exclusively for the bioinformatics processing. The resulting outputs were exported from Galaxy, organized and formatted using Microsoft Excel, and subsequently imported into R Studio for all downstream statistical analyses. Section 2.10 has been revised to clearly state this independent workflow.

Re: Spectrum00692-26R1 (**Comparative Performance of Portable DNA Extraction Protocols and Bioinformatics Workflows for Rapid Detection of Gram-Negative Bacteria and Antimicrobial Resistance Using Oxford Nanopore Sequencing**)

Dear Dr. Michael Frimpong:

Your manuscript has been accepted, and I am forwarding it to the ASM production staff for publication. Your paper will first be checked to make sure all elements meet the technical requirements. ASM staff will contact you if anything needs to be revised before copyediting and production can begin. Otherwise, you will be notified when your proofs are ready to be viewed.

Data Availability: ASM policy requires that data be available to the public upon online posting of the article, so please verify all links to sequence records, if present, and make sure that each number retrieves the full record of the data. If a new accession number is not linked or a link is broken, provide production staff with the correct URL for the record. If the accession numbers for new data are not publicly accessible before the expected online posting of the article, publication may be delayed; please contact ASM production staff immediately with the expected release date.

Publication Fees: For information on publication fees and which article types have charges, please visit ourwebsite. We have partnered with Copyright Clearance Center (CCC) to collect author charges. If fees apply to your paper, you will receive a message from no-reply@copyright.com with further instructions. For questions related to paying charges through RightsLink, please contact CCC at ASM_Support@copyright.com or toll free at +1-877-622-5543. CCC makes every attempt to respond to all emails within 24 hours.

ASM Membership: Corresponding authors may join or renew ASM membership to obtain discounts on publication fees. Need to upgrade your membership level? Please contact Customer Service at Service@asmusa.org.

Embargo Policy: A press release may be issued as soon as the manuscript is posted on theSpectrum Latest Articles webpage. The corresponding author will receive an email with the subject line "ASM Journals Author Services Notification" when the article is available online.

Cover Image Submissions: If you would like to submit a potential Cover Image, please email a file and a short legend to spectrum@asmusa.org. Please note that we can only consider images that (i) the authors created or own and (ii) have not been previously published. By submitting, you agree that the image can be used under the same terms as the published article. Image File requirements: TIF/EPS, 7.5 inches wide by 8.25 inches tall (at least 2,250 pixels wide by 2,475 pixels tall), minimum 300 dpi resolution (600 dpi preferred), RGB, and no figure elements, e.g., arrows or panel labels. The legend should be a short description of the image, 1-2 sentences recommended. Please use the ASM Journals Cover Image Sizing Tool (https://journals.asm.org/pb-assets/html/asm-cover-sizing-tool.html) to ensure that your proposed cover image meets our size requirements.

The ASM Journals program strives for constant improvement in our submission and publication process. Please tell us how we can improve your experience by taking this quick Author Survey.

Thank you for submitting your paper to Spectrum.

Sincerely,
Marisa Nielsen
Editor
Microbiology Spectrum

Reviewer #2 (Comments for the Author):

In the revised manuscript, the authors have inmproved the manuscript by incorporating material that had been supplemental into the main text. This improved the readability of the report and provided easy access to the key data that support the authors' observations. The authors provided appropriate clarifications to the diversity of organisms tested, the calculation of inplementation scores and the cost per test.

Reviewer #3 (Comments for the Author):

The authors addressed all my comments satisfactorily. The only remaining technical point is that the mentioned Bioproject number does not retrieve any data at the moment.
