# Supplementary material for: Comparative performance of portable DNA extraction protocols and bioinformatics workflows for rapid detection of gram-negative bacteria and antimicrobial resistance using Oxford Nanopore sequencing
Source: Microbiol Spectr. 2026 Sep 4;14(10):e00692-26. doi: 10.1128/spectrum.00692-26 (PMC13689490; doi:10.1128/spectrum.00692-26)
Supplement: Supplementary file 2 [file spectrum.00692-26-s0001.pdf]

DNA extraction from Gram-Negative Bacteria using SwiftX DNA Kit.

♦ ***Equipment***

- Appropriate personal protective equipment (PPE)
- Pipettes and disposable pipette tips (aerosol barriers recommended)
- 1.5mL or 2mL microcentrifuge tubes (safe-lock-caps or screwcaps recommended).
- Magnetic stand • Vortexer
- Heating device (water bath, heating block, or thermal shaker)

♦ ***To do before starting***

- Read the complete protocol.
- Heat a water bath, heating block, or thermal shaker to **95°C**.

1. Harvest a sweep of bacterial colony (approximately 2-3 mm in diameter) using a sterile inoculation loop and resuspend it in **700 µl** phosphate-buffered saline (PBS) in a 1.5ml microcentrifuge tube (safe-lock-caps or screwcaps recommended).
2. Centrifuge for **1 minute at 10,000 rpm** to pellet the bacteria. Remove the supernatant.
3. Add **200 µl** of **Buffer DL** to the sample mixture to resuspend the pellets.
4. Shake or vortex **Beads A** for **30 seconds** to ensure homogeneous suspension. Add **30µl** of **Beads A** to the sample mixture. Mix well for 10 seconds by vortexing or inverting the tube.
5. Incubate lysis mixture at **95°C for 10 minutes**. If a thermal shaker is used, shake at maximum speed.
6. Remove sample tube from heating device and mix well for 5 seconds. Remove condensate from the lid before opening by spinning down briefly.
7. Place sample tube into a magnetic stand at room temperature for 1 minute to let the magnetic particles separate.
8. Open the lid while the tube remains in the magnetic stand and transfer the supernatant into a new tube for storage or use in downstream applications.

NB: ***DNA extracts can be stored at -20°C if samples shall be processed later.***

References:

- SwiftX™ DNA-Handbook V1.5.pdf. Xpedite Diagnostics GmbH Lilienthalstr, Germany.
- Schurig, S., Ceruti, A., Wende, A., Lübcke, P., Eger, E., Schaufler, K., Frimpong, M., Truyen, U., Kobialka, R.M. and Abd El Wahed, A., 2024. Rapid Identification of Bacterial Composition in Wastewater by Combining Reverse Purification Nucleic Acid Extraction and Nanopore Sequencing. *ACS ES&T Water*, *4*(4), pp.1808-1818.
- Full Instagene Extraction Protocol_LIT544.pdf. Bio-Rad Laboratories 2000 Alfred Nobel Dr., Hercules, CA 94547 510-741-1000

DNA extraction from Gram-Negative Bacteria using SwiftX DNA Kit + Proteinase K.

- ***Equipment***

• Appropriate personal protective equipment (PPE)
• Pipettes and disposable pipette tips (aerosol barriers recommended)
• 1.5ml or 2ml microcentrifuge tubes (safe-lock-caps or screwcaps recommended).
• Magnetic stand • Vortexer
• Heating device (water bath, heating block, or thermal shaker)

- ***To do before starting***

• Read the complete protocol.
• Heat a water bath, heating block, or thermal shaker to **95°C**.
• Heat a second water bath or heating block to **60°C** for the **Proteinase K incubation** step.

1. Harvest a sweep of bacterial colony (approximately 2-3 mm in diameter) using a sterile inoculation loop and resuspend it in **700 µl** phosphate-buffered saline (PBS) in a 1.5ml microcentrifuge tube (safe-lock caps or screwcaps recommended).

2. Centrifuge for **1 minute at 10,000 rpm** to pellet the bacteria. Remove the supernatant.

3. Add **5µl** of **Proteinase K (20mg/mL)** to the pellet to resuspend, mix again, and incubate at **60°C for 10 minutes**.

4. Add **200 µl** of **Buffer DL** to the sample mixture.

5. Shake or vortex **Beads A** for **30 seconds** to ensure homogeneous suspension. Add **30 µl** of **Beads A** to the sample mixture. Mix well for 10 seconds by vortexing or inverting the tube.

6. Incubate lysis mixture at **95°C for 10 minutes**. If a thermal shaker is used, shake at maximum speed.

7. Remove sample tube from heating device and mix well for 5 seconds. Remove condensate from the lid before opening by spinning down briefly.

8. Place sample tube into a magnetic stand at **room temperature** for **1 minute** to let the magnetic particles separate.

9. Open the lid while the tube remains in the magnetic stand and transfer the supernatant into a new tube for storage or use in downstream applications.
   NB: ***DNA extracts can be stored at -20°C if samples shall be processed later.***

References:

- SwiftX™ DNA-Handbook V1.5.pdf. Xpedite Diagnostics GmbH Lilienthalstr, Germany.
- Schurig, S., Ceruti, A., Wende, A., Lübcke, P., Eger, E., Schaufler, K., Frimpong, M., Truyen, U., Kobialka, R.M. and Abd El Wahed, A., 2024. Rapid Identification of Bacterial Composition in Wastewater by Combining Reverse Purification Nucleic Acid Extraction and Nanopore Sequencing. *ACS ES&T Water*, *4*(4), pp.1808-1818.
- Full Instagene Extraction Protocol_LIT544.pdf. Bio-Rad Laboratories 2000 Alfred Nobel Dr., Hercules, CA 94547 510-741-1000

DNA extraction from Gram-Negative Bacteria using SwiftX ParaBact Kit.

♦ *Equipment*

- Appropriate personal protective equipment (PPE)
- Pipettes and disposable pipette tips (aerosol barriers recommended)
- 1.5ml or 2ml microcentrifuge tubes (safe-lock-caps or screwcaps recommended).
- Magnetic stand • Vortexer
- Heating device (water bath, heating block, or thermal shaker)

♦ *To do before starting*

- Read the complete protocol.
- Heat a water bath, heating block, or thermal shaker to 95°C.

1. Harvest a sweep of bacterial colony (approximately 2-3 mm in diameter) using a sterile inoculation loop and resuspend it in **700 µl** phosphate-buffered saline (PBS) in a 1.5ml microcentrifuge tube (safe-lock-caps or screwcaps recommended).
2. Centrifuge for **1 minute at 10,000 rpm** to pellet the bacteria. Remove the supernatant.
3. Add **200 µl** of **Buffer BPL** to the sample mixture to resuspend the pellets.
4. Shake or vortex **Beads A** for **30 seconds** to ensure homogeneous suspension. Add **30 µl of Beads A** to the sample mixture. Mix well for 10 seconds by vortexing or inverting the tube.
5. Incubate lysis mixture at **95°C for 15 minutes**. If a thermal shaker is used, shake at maximum speed.
6. Remove sample tube from heating device and mix well for 5 seconds. Remove condensate from the lid before opening by spinning down briefly.
7. Open the lid and add **40 µl** of **Buffer AD** to the lysis mixture. Mix well for 5 seconds by vortexing or inverting the tube.
8. Place sample tube into a magnetic stand at **room temperature for 1 minute** to let the magnetic particles separate.
9. Open the lid while the tube remains in the magnetic stand and transfer the supernatant into a new tube for storage or use in downstream applications.

   NB: ***DNA extracts can be stored at -20°C if samples shall be processed later.***

References:

- SwiftX™ Parabact-Handbook V1.3.pdf. Xpedite Diagnostics GmbH Lilienthalstr, Germany.
- Schurig, S., Ceruti, A., Wende, A., Lübcke, P., Eger, E., Schaufler, K., Frimpong, M., Truyen, U., Kobialka, R.M. and Abd El Wahed, A., 2024. Rapid Identification of Bacterial Composition in Wastewater by Combining Reverse Purification Nucleic Acid Extraction and Nanopore Sequencing. *ACS ES&T Water*, *4*(4), pp.1808-1818.
- Full Instagene Extraction Protocol_LIT544.pdf. Bio-Rad Laboratories 2000 Alfred Nobel Dr., Hercules, CA 94547 510-741-1000

Isolation of Genomic DNA from microbial samples using the Macherey-Nagel NucleoSpin Microbial DNA kit.

1. Harvest a sweep of bacterial colony (approximately 2-3 mm in diameter) using a sterile inoculation loop and resuspend it in **700 µl** phosphate-buffered saline (**PBS**) in a 1.5ml microcentrifuge tube (*not provided*).
2. Centrifuge for **3 minutes** at **6,000 x *g*** to pellet the bacteria. **Discard** the supernatant.
3. Add **100 µl** of **Elution Buffer BE** to resuspend the cells. ♦*Alternatively, high-quality molecular-grade water (not provided) can be used*.
4. Transfer the cell suspension into the **MN Bead Tube Type B** (provided).
5. Add **40 µl** of **Buffer MG**. Then, add **10 µl** of **liquid Proteinase K** and close the tube. ♦*It is not necessary to vortex here*.
6. **Agitate** the MN Bead Tube on a swing mill or similar device (e.g. Eppendorf Thermomixer C at **2000 rpm** for **15 min**). ♦ *Optimal agitation duration, speed/frequency depends on the machine used. As a general starting point, disrupt microbial samples for 20 min using the MN Bead Tube Holder on a Vortex Genie® 2. On a Retsch® Mixer Mill MM200, MM300, MM400, e.g., 4 min at maximal frequency (30 Hertz) is adequate for E. coli, 12 min for B. subtilis.*
7. Centrifuge the MN Bead Tube at **11,000 x *g*** for **30 s** to clean the lid. ♦ *In this step, foam is displaced from the screw cap, so that the cap can be removed cleanly.*
8. Add **600 µl** of **Buffer MG** and **mix** (e.g., vortex for 3 s).
9. Centrifuge at **11,000 x *g*** for **30 s.** ♦ *This centrifugation step is performed in order to clean the lid and sediment glass beads and cell debris.*
10. Transfer the **supernatant (~500 – 600 µl)** onto the **NucleoSpin® Microbial DNA Column**, placed in a **2 ml Collection** Tube (*provided*).
11. Centrifuge at **11,000 x *g*** for **30 s**. Discard the collection tube with flowthrough. Put the column into a **fresh 2 ml Collection Tube** (*provided*).
12. Add **500 µl** of **Buffer BW**. Centrifuge at **11,000 x *g*** for **30 s**. **Discard flowthrough** and place the column back into the Collection Tube. (**1st Wash**)
13. Add **500 µl** of **Buffer B5** to the column and centrifuge at **11,000 x *g*** for **30 s**. **Discard flowthrough** and place the column back into the Collection Tube. (**2nd Wash**)
14. Centrifuge the column for **30 s** at **11,000 x *g***. ♦*Residual wash buffer is removed in this step.*
15. Place the **NucleoSpin® Microbial DNA Column** into a **1.5 mL nuclease-free tube** (*not provided*) and add **100 µl** of **Elution Buffer BE** onto the column.
16. **Incubate** at **room temperature** for **1 min**. Centrifuge at **11,000 x *g*** for **30 s.**

NB:
- ***High yield:*** Two serial elutions of **100 µl** each for a total elution volume of **200 µl**.
- ***High concentration:*** Use initial **100 µl** eluate for second elution- **100 µl** total elution volume, 2 elutions.

References

- Macherey Nagel user manual- 12/2022, Rev. 06. MACHEREY-NAGEL GmbH & Co. KG Valencienner Str. 1152355 Düren · Germany. www.mn-net.com
- Schurig, S., Ceruti, A., Wende, A., Lübcke, P., Eger, E., Schaufler, K., Frimpong, M., Truyen, U., Kobialka, R.M. and Abd El Wahed, A., 2024. Rapid Identification of Bacterial Composition in Wastewater by Combining Reverse Purification Nucleic Acid Extraction and Nanopore Sequencing. *ACS ES&T Water*, *4*(4), pp.1808-1818.
