# Supplementary material for: Comparative performance of portable DNA extraction protocols and bioinformatics workflows for rapid detection of gram-negative bacteria and antimicrobial resistance using Oxford Nanopore sequencing
Source: Microbiol Spectr. 2026 Sep 4;14(10):e00692-26. doi: 10.1128/spectrum.00692-26 (PMC13689490; doi:10.1128/spectrum.00692-26)
Supplement: Supplementary file 3 [file spectrum.00692-26-s0004.pdf]

Supplementary Table S1: Scoring rubric and point allocation tally for operational portability and field suitability

Evaluation Criteria	Point Allocation Rubric	SwiftX DNA	SwiftX DNA + ProtK	SwiftX ParaBact	NucleoSpin Microbial
**A. Equipment Portability**	*(Maximum 10 Points)*	**9**	**8**	**9**	**5**
1. Centrifuge Requirement *(maximum 5 points)*	• Portable mini (<5,000 × *g*) = 5 pts<br>• Benchtop microcentrifuge (>10,000 × *g*) = 2 pts	5	5	5	2
2. Thermal Requirement *(maximum 3 points)*	• Simple heat block (shaking optional) = 3 pts<br>• Heat block (strict multi-temp steps) = 2 pts<br>• Dedicated thermal shaker required = 1 pt	3	2	3	1
3. Specialized Hardware *(maximum 2 points)*	• None required = 2 pts<br>• Magnetic rack required = 1 pt	1	1	1	2
**B. Field Suitability & Logistics**	*(Maximum 10 Points)*	**8**	**6**	**9**	**7**
1. Reagent Storage conditions and cold chain dependency *(maximum 4 points)*	• RT kit but 4°C/–20°C after opening = 4 pts<br>• Ambient acceptable (up to 25°C) = 3 pts<br>• 2–8°C preferred (intermediate stability: up to 25 °C ≤8 weeks) = 2 pts<br>• Strict –20°C + chilled transport = 2 pts	2	2	3	4
2. Thermal stability (at 30°C) *(maximum 3 points)*	• Excellent (No efficiency loss) = 3 pts<br>• Good (Minor degradation) = 2 pts<br>• Moderate (Noticeable degradation) = 1 pts	3	1	3	2
3. Workflow complexity *(maximum 2 points)*	• ≤10 steps (Rapid workflow) = 2 pts<br>• >10 steps (Extended workflow) = 1 pt	2	2	2	1
4. Reagent Toxicity & Biosafety *(maximum 1 point)*	• Non-hazardous buffer system (easy field disposal) = 1 pt<br>• Contains hazardous chaotropic salts = 0 pts	1	1	1	0

Scores for each protocol were assigned independently by two authors, with discrepancies resolved by consensus. Thermal stability at 30 °C is representative of tropical/field ambient temperature.

Supplementary Table S2. Kruskal-Wallis Test results comparing mean purity ratios across workflow outcomes

Test	Variable	Groups	H	df	P_value
Kruskal-Wallis	Mean Purity Ratio (A260/A280)	Complete vs. Partial vs. Fail	14.863	2	0.0006

The table presents the non-parametric evaluation of Mean Purity Ratio (A260/A280) differences among three workflow status groups (Complete, Partial, and Fail). H = Kruskal-Wallis test statistic; df = degrees of freedom; P_value indicates statistical significance.

Supplementary Table S3. Dunn’s post-hoc pairwise comparisons of mean DNA purity across workflow outcomes

Comparison	Z_statistic	P_unadjusted	P_adjusted	Significant
Complete - Partial	2.3631	0.0091	0.0272	Yes
Complete - Fail	3.6414	0.0001	0.0004	Yes
Partial - Fail	0.0788	0.4686	1.0000	No

The table presents the results of Dunn's post-hoc test following a significant Kruskal-Wallis test evaluating differences in mean DNA purity (A260/A280 ratio). The P_adjusted column reflects Bonferroni-corrected p-values to account for multiple comparisons. Statistical significance (Yes/No) is determined using the adjusted p-values at an alpha level of 0.05.
