# Supplementary material for: Comparative performance of portable DNA extraction protocols and bioinformatics workflows for rapid detection of gram-negative bacteria and antimicrobial resistance using Oxford Nanopore sequencing
Source: Microbiol Spectr. 2026 Sep 4;14(10):e00692-26. doi: 10.1128/spectrum.00692-26 (PMC13689490; doi:10.1128/spectrum.00692-26)
Supplement: Supplementary file 4 [file spectrum.00692-26-s0003.docx]

**Figure S1: Failure mode distribution across bioinformatics workflow stages.** Bar chart showing the number of isolates failing at each analysis stage (x‑axis) for each extraction protocol (SwiftX DNA, SwiftX DNA + ProtK (proteinase K), SwiftX ParaBact, and NucleoSpin Microbial). Bars represent the frequency of failures per stage and protocol, highlighting widespread multi‑stage failures for SwiftX DNA-based protocols, limited late‑stage failures for SwiftX ParaBact, and the absence of failures for NucleoSpin Microbial.

**Figure S2**: Virulence factor gene counts detected across bacterial isolates by DNA extraction protocol. Bar plots represent the total number of virulence-associated genes identified in each bacterial isolate following DNA extraction using SwiftX ParaBact and NucleoSpin Microbial protocols. Counts are shown above each bar. Across isolates, differences in virulence gene detection were observed between extraction methods, with NucleoSpin Microbial generally yielding higher counts in several isolates, including CC-KAS-001-M1 and the ATCC strain. Minimal variation between protocols was observed for PC-ATO-023-C1.
